# Embryology of the VNO and associated structures in the grass snake *Natrix natrix* (Squamata: Naticinae): a 3D perspective

**DOI:** 10.1186/s12983-017-0188-y

**Published:** 2017-01-13

**Authors:** Paweł Kaczmarek, Mateusz Hermyt, Weronika Rupik

**Affiliations:** Department of Animal Histology and Embryology, University of Silesia, 9 Bankowa Str, 40-007 Katowice, Poland

**Keywords:** Jacobson’s organ, Vomeronasal system, Lacrimal duct, Cupola Jacobsoni, Snake embryos, Evo-devo, 3D reconstruction

## Abstract

**Background:**

Snakes are considered to be vomerolfaction specialists. They are members of one of the most diverse groups of vertebrates, Squamata. The vomeronasal organ and the associated structures (such as the lacrimal duct, choanal groove, lamina transversalis anterior and cupola Jacobsoni) of adult lizards and snakes have received much anatomical, histological, physiological and behavioural attention. However, only limited embryological investigation into these structures, constrained to some anatomical or cellular studies and brief surveys, has been carried out thus far. The purpose of this study was, first, to examine the embryonic development of the vomeronasal organ and the associated structures in the grass snake (*Natrix natrix*), using three-dimensional reconstructions based on histological studies, and, second, to compare the obtained results with those presented in known publications on other snakes and lizards.

**Results:**

Five major developmental processes were taken into consideration in this study: separation of the vomeronasal organ from the nasal cavity and its specialization, development of the mushroom body, formation of the lacrimal duct, development of the cupola Jacobsoni and its relation to the vomeronasal nerve, and specialization of the sensory epithelium. Our visualizations showed the VNO in relation to the nasal cavity, choanal groove, lacrimal duct and cupola Jacobsoni at different embryonic stages. We confirmed that the choanal groove disappears gradually, which indicates that this structure is absent in adult grass snakes. On our histological sections, we observed a gradual growth in the height of the columns of the vomeronasal sensory epithelium and widening of the spaces between them.

**Conclusions:**

The main ophidian taxa (Scolecophidia, Henophidia and Caenophidia), just like other squamate clades, seem to be evolutionarily conservative at some levels with respect to the VNO and associated structures morphology. Thus, it was possible to homologize certain embryonic levels of the anatomical and histological complexity, observed in the grass snake, with adult conditions of certain groups of Squamata. This may reflect evolutionary shift in Squamata from visually oriented predators to vomerolfaction specialists. Our descriptions offer material useful for future comparative studies of Squamata, both at their anatomical and histological levels.

**Electronic supplementary material:**

The online version of this article (doi:10.1186/s12983-017-0188-y) contains supplementary material, which is available to authorized users.

## Background

Complete vomeronasal system (VNS or accessory olfactory system) has been found in lungfish [1–3] amphibians [4–7], reptiles [8–10] and mammals [10]. It is formed by the sensory epithelium, accessory olfactory bulbs and vomeronasal- recipient areas of the telencephalon [11, 12]. A growing body of evidence suggests that secretions of the Harderian gland, drained by the lacrimal or nasolacrimal duct, are linked with the chemoreceptive function of the vomeronasal organ. Hence, these two additional components should be considered as parts of the VNS [13–16]. In comparison to the main olfactory system, the vomeronasal system tends to be sensitive to less-volatile molecules and the function of vomerolfaction is considered in the context of location and discrimination of prey, avoidance of predator and reproduction [11, 17].

**Fig. 1:**
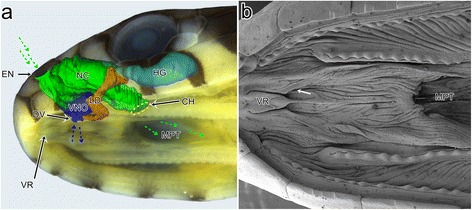
**a** The scheme of the left side of the adult-like ophidian snout showing the spatial relationships of the vomeronasal organ, the nasal cavity, lacrimal duct, the Harderian gland and the palate (according to material obtained in present study and description provided by Parsons [23]. The main olfactory system tend to be sensitive to the volatile molecules (*green arrows*) in contrast to less volatile molecules (*blue arrows*) detected by the accessory olfactory system. **b** Scanning electron micrograph of palate of the grass snake embryo at developmental stage XI. *White* arrow indicates the putative position of the palatal opening of the left vomeronasal organ duct. Abbreviations: *CH* choana, *DV* duct of the vomeronasal organ, *EN* external naris, *HG* Harderian gland, *LD* lacrimal duct, *NC* nasal cavity, *MPT* median palatal trough, *VNO* vomeronasal organ, *VR* vomerine raphe

The vomeronasal organ (VNO), otherwise known as the Jacobson’s organ, is a patch of microvillar chemosensory epithelium that may take different forms such as a sac or a duct [18]. The VNO is usually associated with the non-sensory ciliated epithelium, which is sometimes termed the receptor-free epithelium (RFE) [8, 19–21]. Generally, the sensory vomeronasal epithelium can be distinguished from the olfactory epithelium by three main homologues – lack of the Bowman’s glands, association of the nerve of the VNO with the accessory olfactory bulb (not main olfactory bulb), and ventral (not dorsal) location of the organ [22]. The VNO of snakes and lizards, except for chameleons, is well developed and contains the sensory epithelium of the dorsal dome and the non-sensory epithelium that lines the ventral concha, which is called the mushroom body. The paired VNOs communicate with the oral cavity through two ducts which open separately by means of two palatal openings that are located anteriorly to the internal nares or choanae which in snakes enter the median palatal trough (Fig. 1a, b) [22–26]. In Squamata, the lacrimal or nasolacrimal duct discharges directly into the duct of the VNO, as in snakes, or is connected with the choanal groove, as in most lizards [23, 24, 27].

In Squamata, the developing VNO is connected to the forming nasal cavity, and then becomes gradually separated from the latter [23, 24, 28]. In adults of this clade, the nasal cavity consists of three main parts: the vestibulum, conected with the external naris, the main nasal cavity, and the nasopharyngeal duct leading to the choana. The main nasal cavity is subdivided into the anterior caval zone, conchal zone (with single concha) and Antorbitalraum. The concha divides the conchal zone into the lateral and dorsolateral Sakter, dorsomedial Stammteil, and ventromedial Choanengang [23].

The nasal cavity of birds and nonavian reptiles (excluding crocodilians) forms in their embryos as a groove between the medial nasal prominence of the frontonasal mass and the lateral parts of the primary palate (the lateral nasal and maxillary prominences). The lateral nasal prominences is separated from the maxillary prominences by the nasolacrimal groove [29–31].

The VNO in squamate reptiles (excluding Iguania) is enclosed in its bony enclosure called the cupola Jacobsoni [32]. This structure is generally well developed in snakes. In this group, the cupola Jacobsoni is formed by only two bones – the vomer and septomaxilla [32–34]. Each of the paired vomers of advanced snakes (Caenophidia) consists of the vertical plate of the triangular shape with a large foramen on its posterior part. The globular cup, completed by the septomaxilla, is attached to the middle of the plate. Anteriorly, the septomaxilla creates a rod-like structure, then extends posteriorly as a slightly dome-shaped roof over the VNO and terminates on its caudal end as a hook-shaped, condylar surface. The characteristic dorsal process emerges from the lateral part of the septomaxillary plate [33–35].

The VNS and cupola Jacobsoni of adult squamates have received much anatomical (e.g. [24, 27, 31–43]), histological (e.g. [8, 19, 44–48]), physiological (e.g. [16, 49–52]) and behavioural (e.g. [9, 52–54]) attention. It has, however, received only a limited embryological investigation, constrained to some anatomical [23, 24, 28, 31] or cellular studies [29, 55, 56] and brief surveys [57].

The purpose of this study was to examine the embryonic development of the VNO, lacrimal duct (mostly its rostral aspect) and other associated structures (the nasal cavity or choanal groove and, at later stages of their development, the lamina transversali anterior and cupola Jacobsoni) of the grass snake, *Natrix natrix* (Squamata: Natricinae) using three-dimensional reconstructions based on our histological studies. It is worth mentioning that experimental data, such as histological images, provide only partial static information about the organogenesis of any organ [58]. That is why we applied tools that integrate these separate individual pieces of data into a 3D model. Moreover, the obtained sections were used to analyse the histology of the sensory epithelium of the VNO and structures that were reconstructed. Thus, the anatomical and histological descriptions as well as 3D images provided by us were used for comparisons to known publications on other snakes and squamates. Indeed, different levels of anatomical or histological complexity, observed in the grass snake embryos in different organs, resemble adult structures of other non-ophidian squamates or rhynocephalians (*Sphenodon*). Thus, we proposed potential homologues in their evolution. We believe that this paper provides an important contribution to the understanding of the formation and evolution of the VNO and associated structures in Squamata and could serve as a basis for further comparative studies.

## Methods

### Animal and embryo manipulation

Fertilised female grass snakes (*N. natrix*) were caught in Poland, in the vicinity of Wroclaw and Lubliniec, at the beginning of June in 2014 and 2015, respectively. In agreement with the granted permissions, two females were caught each year, thus a total of 4 females were acquired for this study. In total, 60 eggs were obtained. The animals were kept in vivaria, in conditions similar to those in the wild, until their eggs were laid and they were then released into their native areas.

The grass snake eggs were incubated at 30 °C at a relative humidity of 100% in a small chick incubator. The eggs were half buried in a substrate composed of a sand and peat mixture at 1:1 ratio and stored in plastic food storage containers with clear, see-through sides and tops. The depth of the substrate was ca. 10 cm and the air layer above it was ca. 5 cm. Holes in the bottom and top of the containers ensured air circulation. Embryonic development, at 30 °C under laboratory conditions from the time the eggs were laid until they hatched, lasted 30–33 days. The embryos used for examination were isolated at regular intervals (Journal of Laws, 2005 No. 33 item 289), starting immediately after the eggs were laid and ending when the first individuals hatched (Table 1).

**Table 1:** Obtained developmental stages of the grass snake and the numbers of isolated embryos, performed sets of serial section and 3D reconstructions

Days post oviposition (d.p.o.)	Developmental stage	No of isolated embryos (viable)	No of sets of serial sections	No of 3D reconstructions
2–3	II	2	2	1
5–6	IV	4	4	2
8	V	4	4	1
10–13	VI	5	4	1
18	VIII	5	5	1
24	X	2	1	0
27–28	XI	5	4	1
Total		27	24	7

The research was performed from 2014 to 2016. The age of the embryos was calculated using the *N. natrix* developmental table by Rupik [59], which fulfils all the criteria for standard developmental tables. The relationship between the incubation time and embryo body size is presented in Table 2 [59].

**Table 2:** The relationship between the incubation time and the embryo body size

Days post oviposition (d.p.o.)	Total length (mm)	Head length (mm)	Head width (mm)	Head height (mm)	Tail length (mm)	Embryo age defined by stages
0	26.5	2.3	1.4	3.9	No	I
1	27.0	2.5	1.6	4.4	No	
2	30.0	3.2	1.8	4.8	No	II
3	31.5	3.7	1.8	4.9	2.0	
4	32.1	3.9	2.0	4.5	3.0	III
5	34.9	3.9	2.1	4.3	4.3	IV
6	43.8	4.1	2.2	4.2	5.2	
7	48.7	4.5	2.4	4.2	5.7	V
8	58.5	4.8	2.4	4.0	7.8	
9	79.3	4.8	2.5	4.0	9.2	
10	82.7	5.2	2.7	3.8	11.3	VI
12	87.6	6.4	2.7	3.7	11.9	
14	98.2	7.1	2.9	3.7	15.8	VII
17	133.7	8.0	3.3	3.5	17.1	VIII
20	144.2	8.3	3.8	3.3	25.9	IX
22	153.1	8.7	3.9	3.2	29.3	X
25	176.3	9.4	4.0	3.0	30.7	XI
27	184.7	11.3	4.2	3.0	34.2	
31	200.0	11.8	4.7	3.0	38.5	XII
32	211.3	12.0	4.7	3.0	40.0	
33	211.8	12.0	4.7	3.0	40.1	

From Rupik [59]

### Light microscopy technique

The heads of the grass snake embryos were fixed in 10% formalin and in Bouin’s fluid, then dehydrated and paraffin-embedded using standard methods [60]. Transverse section series were cut at 7 μm using a Leica rotary microtome (Leica RM2125RT). Paraffin sections were stained using the AZAN method, after Heidenhain [60], and with Ehrlich’s haematoxylin and eosin [61, 62]. All histological sections (7-μm thick) were sequentially photographed, using a light microscope OLYMPUS BX60 with an OLYMPUS DP12 digital camera, and archived as TIFF files, using CellSens Standard software. The number of sets of serial sections was shown in Table 1.

### 3D reconstructions

One set of serial sections (see above) of the best quality was used for most obtained stages (Table 1). Three-dimensional reconstructions were carried out for the VNO and associated structures, such as the lacrimal duct, lamina transversali anterior, vomer, septomaxilla, choanal groove and, at the earliest stages, nasal cavity of the grass snake embryos. The TrakEM2 plug-in of ImageJ software (NIH, USA) was used to align the images in image stacks. Images were organised in the correct order (from the top to the bottom) in the image stacks according to the image numbers. Image distances were designated between the sections. The borders of the VNO and associated structures were obtained using the surface creation tool of this program, which calculates the contour surfaces of reconstructed organs based on the contour lines that are defined manually. In the case of the VNO, the nasal cavity and the lacrimal duct, the reconstructed areas contained the lumens of the organs and their epithelia. The contour lines for each visualised structure were placed independently using different colours. Each surface could be shown separately or be accompanied by other surfaces. ImageJ is a freely available software package.

### Scanning electron microscopy (SEM)

To show the general morphology of the palate in the grass snake (Fig. 1b), archival material (the grass snake embryo at developmental stage XI), prepared for another scanning electron microscopic study, has been used. This material was fixed in a 1:1 mixture of 2.5% glutaraldehyde and 2% paraformaldehyde in 0.1 M phosphate buffer (pH 7.4) at 4 °C for 24 h. After rinsing in phosphate buffer the samples were dried using a Pelco CPD critical point dryer, gold-coated using a PELCO SC-6 coater and examined in a Hitachi UHR FE-SEM SU 8010 scanning electron microscope (Laboratory of Scanning Microscopy, Faculty of Biology and Environmental Protection, University of Silesia).

## Results

On the basis of our histological analysis and 3D reconstructions, we were able to distinguish five major concurrent developmental processes occurring during the development of the VNO and associated structures: 1. separation of the VNO from the nasal cavity and its specialization, 2. development of the mushroom body, 3. formation of the lacrimal duct, 4. development of the cupola Jacobsoni and its relation to the vomeronasal nerve, 5. specialisation of the vomeronasal sensory epithelium.

### 1. Separation of the VNO from the nasal cavity and its specialization

#### Stage II

**Fig. 2:**
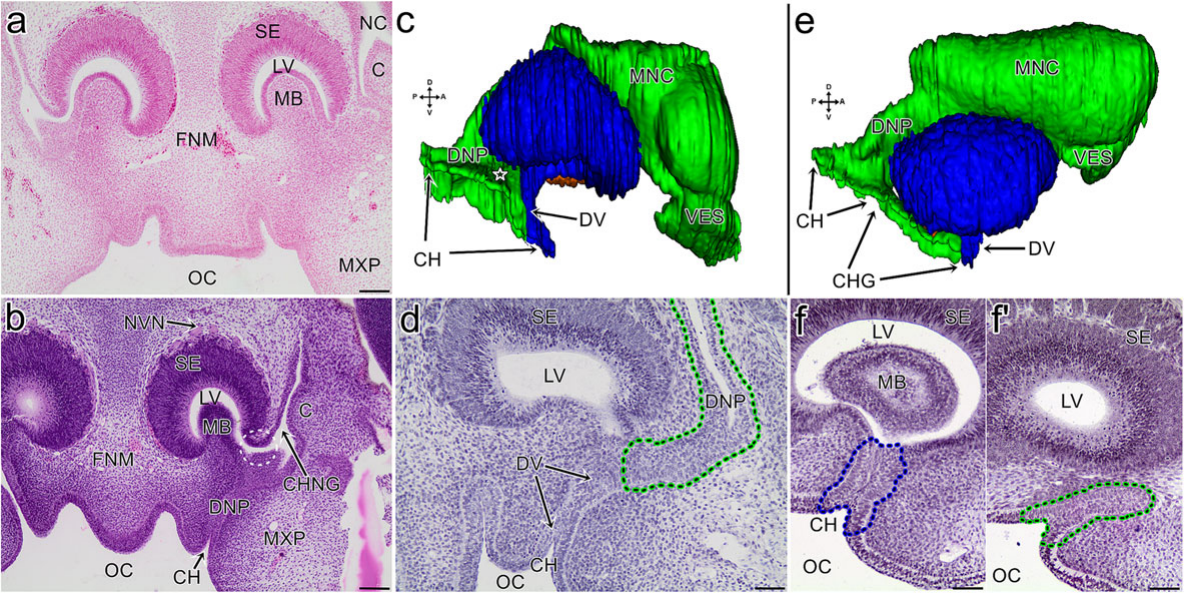
Transverse sections (**a**, **b**, **d**, **f** and **f**’) through the snout and 3D reconstructions (**c** and **e**) of the vomeronasal organ and associated structures of the grass snake embryos at different developmental stages. **a** The vomeronasal organ at the end of developmental stage II. **b** The vomeronasal organ at the end of developmental stage II. The *dashed line* indicates the connection of the VNO with the nasal cavity. **c** The medial aspect of the vomeronasal organ (*blue*) and its relation to the nasal cavity (*green*) and the lacrimal duct (*orange*) at the beginning developmental stage IV. The *star* indicates the area of the future fusion between the maxillary process and the frontonasal mass. **d** The connection between the primordium of the vomeronasal organ and the developing nasopharyngeal duct at the beginning of developmental stage IV. **e** The medial aspect of the vomeronasal organ and its relation to the nasal cavity and the lacrimal duct at developmental stage V. The colours of the structures are the same as in the c. **f** The embryonic vomeronasal organ and its duct (*blue dashed line*) at developmental stage V. **f’** The choanal groove (*green dashed line*) at developmental stage V. Seven sections posterior to f. Abbreviations: *C* concha of the nasal cavity, *CH* choana, *CHG* choanal groove, *CHNG* Choanengang, *DNP* nasopharyngeal duct, *DV* duct of the vomeronasal organ *FNM* frontonasal mass, *LV* lumen of the vomeronasal organ, *MB* mushroom body, *MNC* main nasal cavity, *MXP* maxillary process, *NC* nasal cavity, *NVN* vomeronasal nerve, *OC* oral cavity, *SE* sensory epithelium of the vomeronasal organ, *VES* vestibulum. Scale bars 100 μm (**a**, **b**); 50 μm (**d**, **f** and **f’**). 3D reconstructions are not scaled

In the earliest grass snake embryos that were analysed, the medial prominence of the frontonasal mass and the lateral nasal prominence were fused. Thus, the primordia of the choanae and external nares were separated and the vestibulum was formed. The remaining posterior part of the embryonic nasal cavity was well developed and largely separated from the forming oral cavity due to the fusion between the anterior part of the maxillary prominence and the frontonasal mass. The main nasal cavity with a well-developed concha was clearly distinguishable. The anlage of the nasopharyngeal duct entered into the oral cavity via the primitive slit-like choana which was located posteriorly between the unfused frontonasal mass and the maxillary prominence. The differentiating VNO exhibited a spherical shape and was located ventromedially to the main nasal cavity, within the tissue of flat frontonasal mass (Fig. 2a). The shape of the latter was almost entirely obliterated because of the aforementioned fusion of the facial prominences (medial, lateral and maxillary). Only a small flat prominence was present at the level of the anterior portion of the choana, passing into the medial ridge more posteriorly (Fig. 2a, b). At this stage, the developing VNO was still connected with the embryonic nasal cavity via a patent duct. It entered the latter between the primordium of the Choanengang and the nasopharyngeal duct anlage (see Fig. 2b- dashed line).

#### Stages IV and V

At the beginning of the developmental stage IV, the anlage of the VNO and the nasal cavity increased in size. The conchal zone and the rest of the posterior part of the nasal cavity created a recess that covered the anterolateral part of the VNO. Due to the growth of the nasal cavity, the VNO lost its connection to the Choanengang and it was now connected only to the nasopharyngeal duct via the forming definitive duct of the VNO, which developed from the anterior part of the nasopharyngeal duct. The nasopharyngeal duct led to the slit-shaped choanae and was patent, except for the area of the forming VNO duct (Fig. 2c, d). The general shape of the VNO did not exhibit significant differences.

At the end of this developmental stage and at the beginning of stage V, the anterior portion of the choana, just behind the developing duct of the VNO, became the choanal groove. It was a result of the choana’s separation from the nasopharyngeal duct by the fusion between the maxillary process and the frontonasal mass in this area. More posteriorly, this fusion was absent, and the choanal groove was continuous with the choana, restricted in length at that time (Fig. 2e, f, f’). The choanal groove was lined by the columnar epithelium and the medial and lateral walls were in apposition to each other. Thus, only the most ventral part of the groove was patent. There were no cartilages or bones that supported this structure (see Fig. 2f’).

The differentiating VNO became slightly elongated in the longitudinal axis. It entered the embryonic oral cavity via its own duct and its ventral part represented the anterior part of the choanal groove at that time. Thus, the nasal cavity and VNO were now connected only by the choanal groove (see Fig. 2e). The duct of the VNO left the posteroventral part of the organ lumen, but it was occluded (see Fig. 2f).

#### Stage VI

The VNO was now a distinct structure from the nasal cavity. At the beginning of this stage, the choanal groove extended on the palate from the ventral part of the duct of the VNO and terminated posteriorly at some distance before the choana. In their anterior course, the choanae entered the palate separately, as previously, but they communicated dorsally and terminated more posteriorly in the deep medial palatal trough (Additional file 1).

**Fig. 3:**
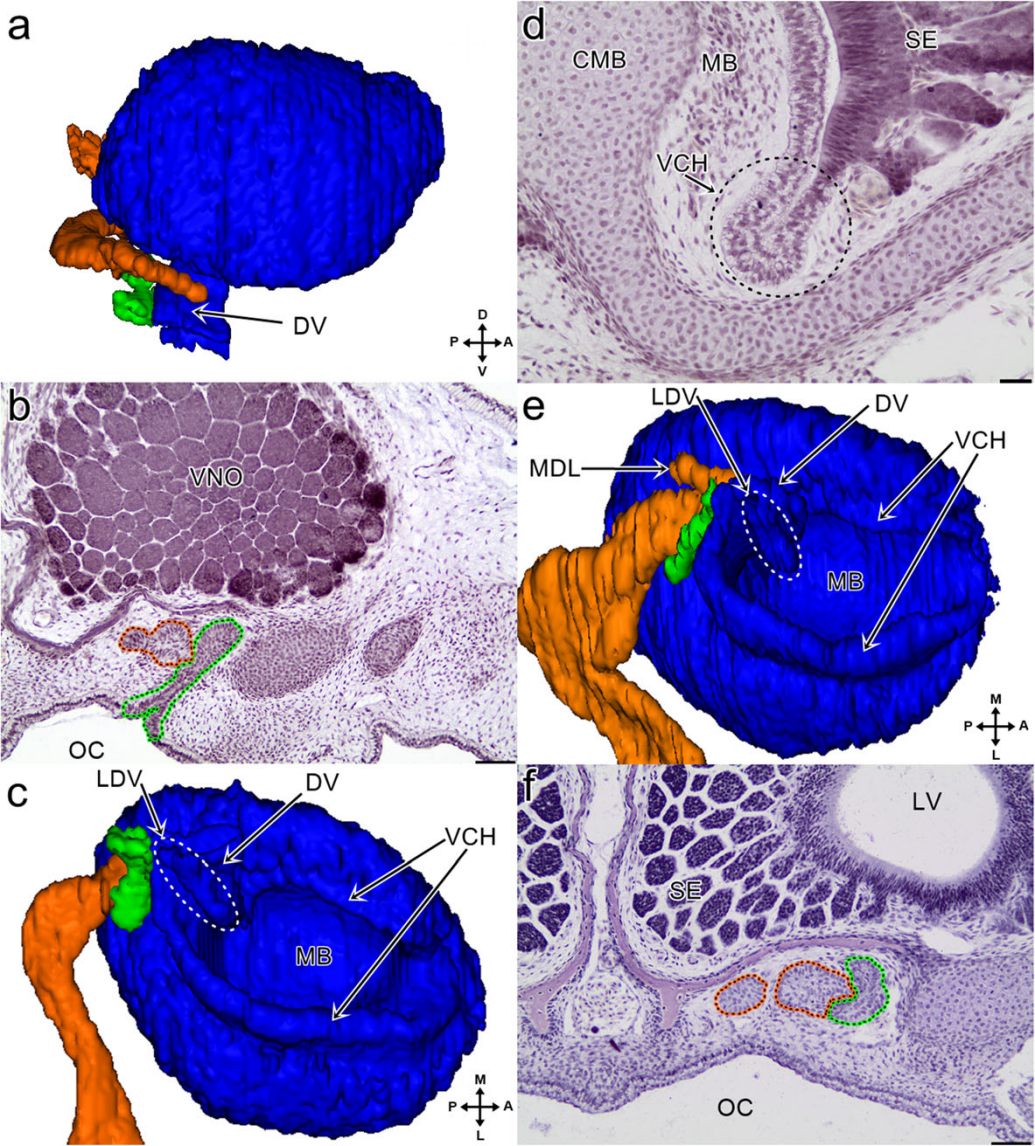
3D reconstructions (**a**, **c** and **e**) of the vomeronasal organ and associated structures and transverse sections (**b**, **d** and **f**) through the snout of the grass snake embryos at different developmental stages. **a** The medial aspect of the vomeronasal organ (*blue*) and its relation to the vestige of the choanal groove (*green*) and the lacrimal duct (*orange*) at the developmantal stage VI. **b** The posterior part of the vomeronasal organ at developmental stage VI. The section at the level of the vestige of the choanal groove (*green dashed line*) connected with the lacrimal duct (*orange dashed line*). **c** The ventral aspect of the vomeronasal organ at developmental stage VI. The colours of the structures are the same as in the a. **d** The lateral part of the ventral channel (*indicated by black dashed line*) at developmental stage VI. **e** The ventral aspect of the vomeronasal organ at developmental stage VIII. The colours of the structures are the same as previously. **f** The lacrimal duct and the vestige of the choanal groove at developmental stage VIII. The colours of the dashed line are the same as in 3b. Nine sections posterior to the level of the connection of the vestige of the choanal groove to the palate. Abbreviations: *A* anterior, *CMB* cartilage of the mushroom body, *D* dorsal, *DV* duct of the vomeronasal organ, *L* lateral, *LDL* lateral diverticulum of the lacrimal duct, *LDV* lateral eminence of the VNO duct, *LV* lumen of the vomeronasal organ, *M* medial, *MB* mushroom body, *MDL* medial diverticulum of the lacrimal duct, *OC* oral cavity, *P* posterior, *SE* sensory epithelium, *V* ventral, *VCH* ventral channel, *VNO* vomeronasal organ. Scale bars 50 μm (**b** and **f**); 20 μm (**d**). 3D reconstructions are not scaled

At the end of this stage, the choanal grove was almost entirely absent. However, the small anterior recess located just posterior to the duct of the VNO appeared to be a remnant of the choanal groove (Fig. 3a, b). It is worth mentioning that the posterior communication of the nasal cavities to each other increased and, before entering the oral cavity, the nasopharyngeal ducts joined together (Additional file 1).

The VNO remained elongated but its medial wall became considerably flattened. The lumen of the organ developed ventrally around the base of the mushroom body. At the end of this stage, this created a distinct ventral channel, which is probably homologous to the spiral channel in lizards described by Pratt [29] (Fig. 3c, d). The channel extended from the anterodorsal part of the duct of the VNO and ran around the base of the mushroom body reaching again the duct from its posterolateral and dorsal part. After leaving the duct, a short section of the medial part of the channel sloped ventrally and then became nearly horizontal and remained so until it reached the anterior part of the VNO. In this part, it bent dorsally so that its shape was of an inverted letter U. The remaining part of the ventral channel sloped ventrally towards the second connection to the duct of the VNO. It was also the most pronounced part of the spiral channel. It is worth mentioning that there were no definitive borders between the ventral channel and the duct of the VNO or between the latter and the residue of the choanal groove. Thus, the extension of all these structures could only be assessed on the basis of their external morphology (see Fig. 3c).

At the end of this stage, the duct of the VNO left the posteromedial part of the organ cavity and was directed medially. Externally, it had a flattened tube shape with a cylindrical eminence on the lateral side that ran obliquely from the posteromedial end of the spiral channel to the posterior part of the entrance of the VNO into the oral cavity (see Fig. 3c- LDV, dashed line). It was still nonpatent (not shown).

#### Stage VIII

The VNO was now more elongated due to the development of the posterior part of the sensory epithelium. The contact of the vestigial choanal groove with the palate became reduced, but it was still present. The dorsal part of this vestige was elongated posterolaterally and ran along the lacrimal duct for a short distance (Fig. 3e, f). The lateral eminence of the VNO duct was now well pronounced and became more vertical rather than oblique (see Fig. 3e- LDV, dashed line).

#### Stage X

The patency of the VNO duct was well visible, except for its lateral eminence (not shown).

#### Stage XI

**Fig. 4:**
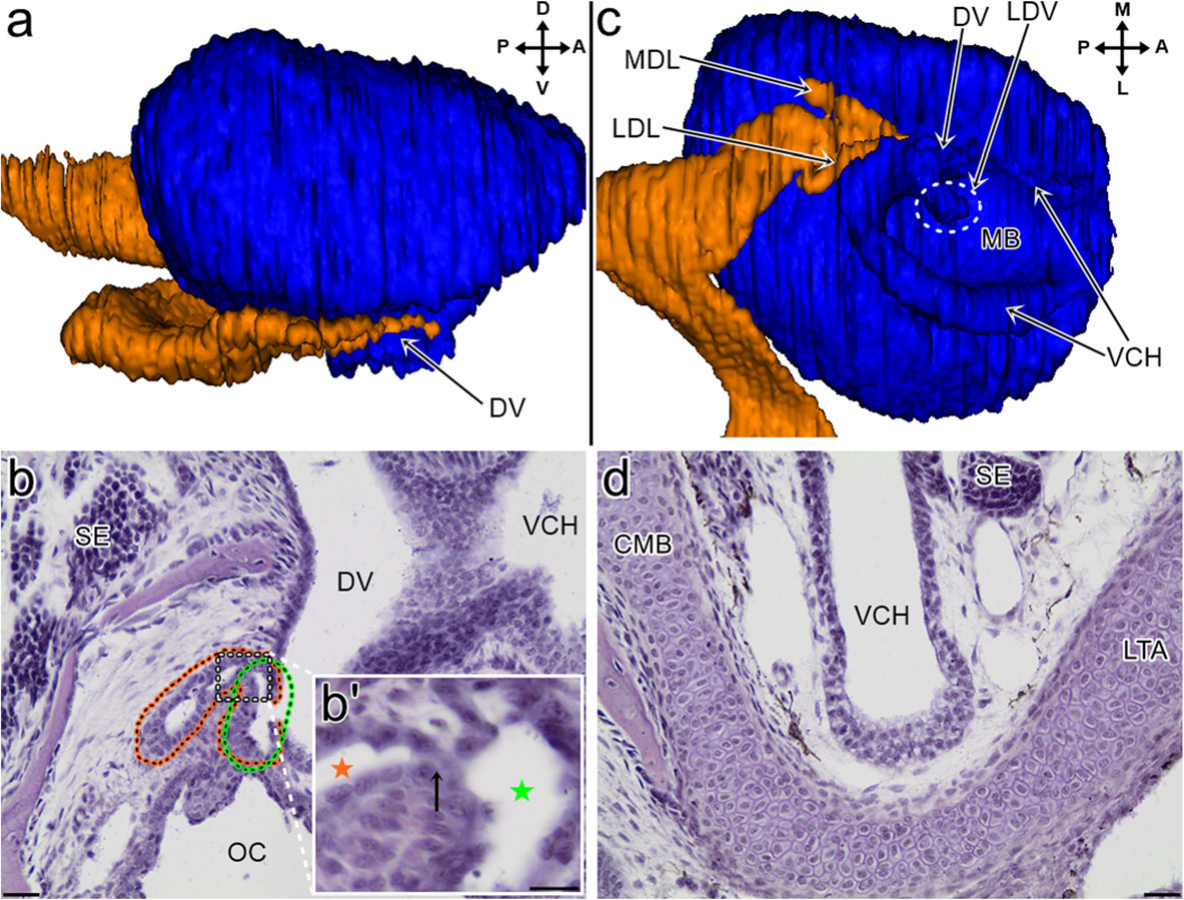
3D reconstructions (**a** and **c**) of the vomeronasal organ and associated structures and transverse sections (**b** and **d**) through the snout of the grass snake embryos at developmental stage XI. **a** The medial aspect of the vomeronasal organ (*blue*) and its relation to the lacrimal duct (*orange*). **b** The connection of the lacrimal duct (*orange dashed line*) with the duct of the vomeronasal organ through the lateral diverticulum of the lacrimal duct (*green dashed line*). **b’** The “connection” (*arrow*) between the lumen of the “orginal” lacrimal duct (*orange star*) and the lateral diverticulum of the lacrimal duct (*green star*). Magnification of the corresponding place marked by the frame in 4b, four sections posteriorly. **c** The ventral aspect of the vomeronasal organ and the rostral part of the lacrimal duct. The colours of the structures are the same as in the a. **d** The lateral part of the ventral channel. Abbreviations: *A* anterior, *CMB* cartilage of the mushroom body, *D* dorsal, *DV* duct of the vomeronasal organ, lateral eminence of the vomeronasa organ duct, *L* lateral, *LDL* lateral diverticulum of the lacrimal duct, *LDV* lateral eminence of the VNO duct, *LTA* lamina transversalis anterior, *M* medial, *MB* mushroom body, *MDL* medial diverticulum of the lacrimal duct, *OC* oral cavity, *P* posterior, *SE* sensory epithelium, *V* ventral, *VCH* ventral channel. Scale bars 20 μm (**b** and **d**); 10 μm (**b’**). 3D reconstructions are not scaled

The dorsal dome of the sensory epithelium was well developed. The medial wall of the VNO was extended ventrally, and thus, in the medial view, it covered the majority of the duct of the VNO and only the ventral part of this duct was visible in this aspect (Fig. 4a). The residue of the choanal groove is patent and it entered the ventral part of the VNO duct rather than the palate, and therefore (and because of the close spatial association with the lacimal duct- see above) it could now be considered to be the lateral diverticulum of the anterior part of the lacrimal duct (Fig. 4b- green dashed line- and Fig. 4c). However, the patency between the “orginal” lacrimal duct and the lateral diverticulum seemed to be very narrow or absent (Fig. 4b’- black arrow). The lateral eminence of the duct of the VNO increased in size and its most ventrolateral part turned anteriorly (see Fig. 4c- LDV, dashed line). The lumen of the duct of the VNO, except for the still largely occluded lateral eminence, was wider now (not shown). Externally, the medial part of the ventral channel was almost entirely obliterated (see Fig. 4c). Its lateral part became significantly larger (Fig. 4d and see Fig. 4c).

### 2. Development of the mushroom body

#### Stage II

The embryonic mushroom body was distinguishable and reduced the lumen of the organ to an inverted bowl-like space. There was no cartilaginous support for this concha (see Fig. 2b).

#### Stage IV

**Fig. 5:**
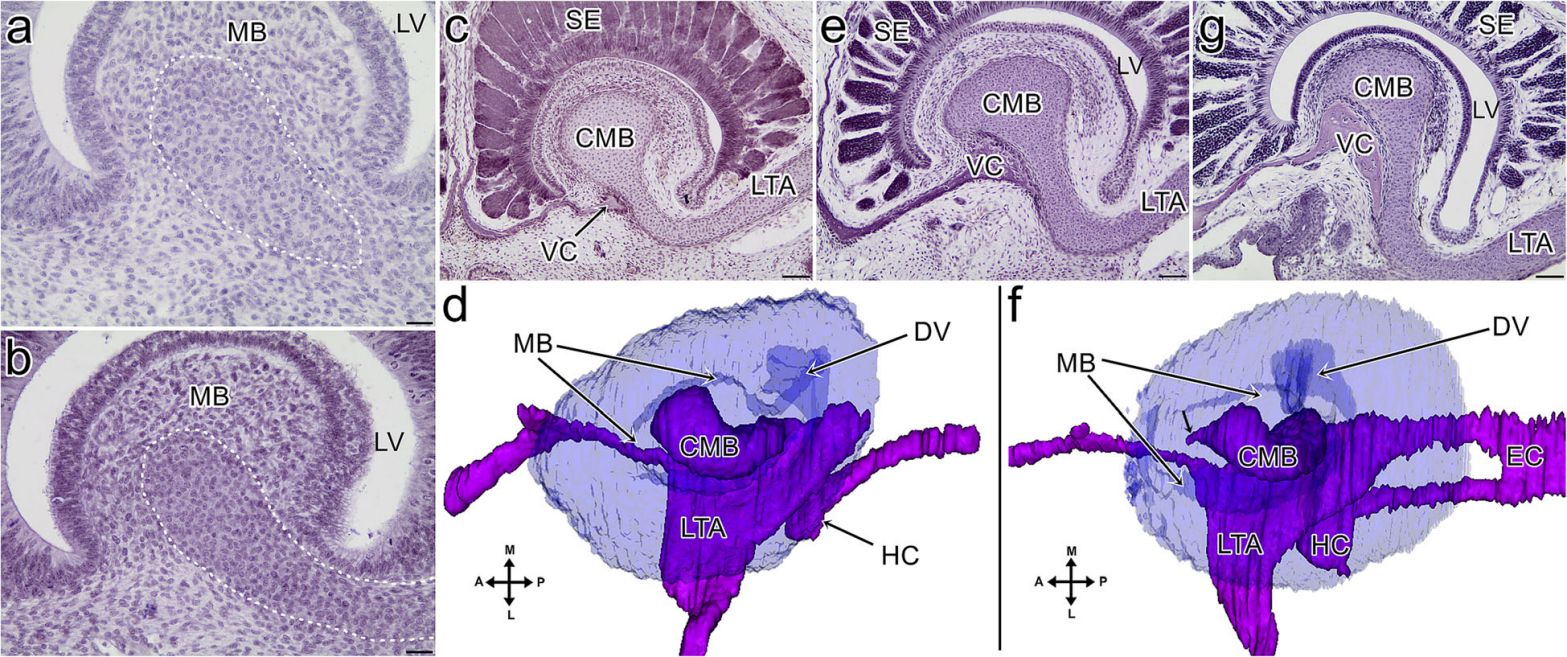
Transverse sections (**a-c**, **e** and **f**) and 3D reconstructions (**d** and **f**) of the mushroom body cartilage in the grass snake embryos at different developmental stages. **a** The condensed mesenchymal cell (*dashed line*) at developmental stage IV. **b** The condensed mesenchymal cell (*dashed line*) at developmental stage V. **c** The cartilage of the mushroom body at the end of developmental stage VI. **d** The dorsal aspect of the mushroom body cartilage and associated cartilages (*purple*) at developmental stage VI. Note that the vomeronasal organ (*blue*) is transparent to show the mushroom body. **e** The cartilage of the mushroom body at developmental stage VIII. **f** The dorsal aspect of the mushroom body cartilage and associated cartilages at developmental stage VIII. Short arrow indicates the conical process of the mushroom body cartilage. The colours of the structures and transparency are the same as in d. **g** The cartilage of the mushroom body at developmental stage XI. Abbreviations: *A* anterior, *CMB* cartilage of the mushroom body, *DV* duct of the vomeronasal organ, *EC* ectochoanal cartilage, *HC* hypochoanal cartilage, *L* lateral, *LTA* lamina transversalis anterior, *LV* lumen of the vomeronasal organ, *M* medial, *MB* mushroom body, *P* posterior, *SE* sensory epithelium of the vomeronasal organ, *VC* vomerine concha. Scale bars 50 μm (**c**, **e**, **g**); 20 μm (**a** and **b**). 3D reconstructions are not scaled

The mushroom body primordium did not exhibit significant differences. At the end of this developmental stage, within the primordium of the mushroom body, the anlage of the mushroom body cartilage was visible as a centrally located condensed mesenchymal cells (Fig. 5a).

#### Stage V

The condensed mesenchymal cell area of the mushroom body cartilage primordium was more visible on the histological sections and was clearly connected to the rest of the forming lamina transversalis anterior, which was located outside the VNO (Fig. 5b).

#### Stage VI

The lumen of the VNO cavity was almost filled due to the development of the mushroom body (Fig. 5c). In fact, the lumen was mainly visible anteriorly. The mushroom body gained cartilaginous support, that is the dorsal projection of the lamina transversalis anterior (Fig. 5d and see Fig. 5c).

The cartilage of the mushroom body and the vomerine parts were separated only by a thin layer of connective tissue (see Fig. 5c). The cartilage of the mushroom body was broadened dorsally, mostly anterodorsally. From a dorsal aspect, the broadened dorsal part resembled a U-like structure with the convex part that was directed laterally and where the anterior rami of this structure was more expanded than the posterior one (see Fig. 5d).

The base of the mushroom body cartilage was supported anteromedially by an extension of the vomer called the vomerine concha (see Fig. 5c). This bony support terminated posteriorly before the duct of the VNO.

#### Stage VIII

The lumen of the VNO appeared to be slightly wider, probably due to the overall growth of the entire organ (Fig. 5e). The vomerine support of the anteromedial part of the base of the cartilage was more massive and developed along the dorsoventral axis (see Fig. 5e). It terminated posteriorly just before the duct of the VNO. The posterior part of the vomerine concha was closer to the VNO duct.

In comparison with the previously described stage, the mushroom body was extended more anteriorly. At this stage, the conical process, which was directed anteriorly, emerged from the broadened anterodorsal part of the cartilage of the mushroom body (Fig. 5f- short arrow). The posterior part of the base of the mushroom body cartilage was bent significantly medially (see Fig. 5f).

#### Stage XI

The lumen of the VNO became significantly larger. The vomerine support of the cartilage of the mushroom body was also well developed. Posteriorly, it almost reached the dorsal part of the cartilage (Fig. 5g).

### 3. Formation of the lacrimal duct

#### Stage II

**Fig. 6:**
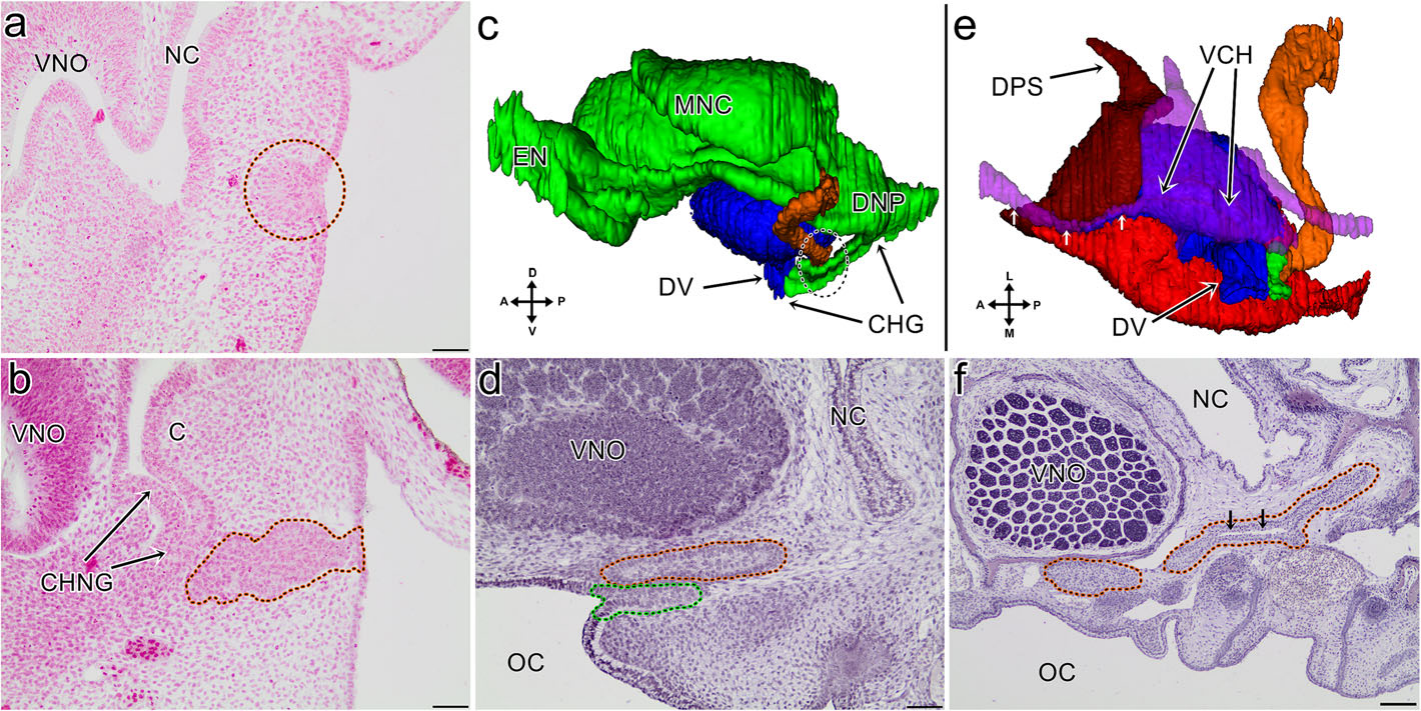
Transverse sections (**a**, **b**, **d** and **f**) and 3D reconstructions (**c** and **d**) of the lacrimal duct in the grass snake embryos at the different developmental stages. **a** The bud of the lacrimal duct (indicated by *orange dashed line*) at the beginning of developmental stage II. **b** The lacrimal duct (*orange dashed line*) in apposition to the Choanengang at the end of developmental stage II. **c** The lacrimal duct (*orange*) in relation to the vomeronasal organ (*blue*), nasal cavity and choanal groove (*green*) at developmental stage V. **d** The lacrimal duct (*orange dashed line*) in relation to the choanal groove (*green dashed line*) at developmental stage V. The section at the level of area indicated by the dashed line on c. **e** The ventral aspect of the lacrimal duct (*orange*) in relation to the vomeronasal organ (*blue*), the vestige of the choanal groove (*green*), associated cartilages (*transparent purple*; *short arrows* indicated the cylindrical part of the lamina transversalis anterior) the vomer (*bright red*) and the septomaxilla (*dark red*) at developmental stage VI. **f** The restricted areas of patency (*short black arrows*) of the lacrimal duct (*orange dashed lines*) at developmental stage VIII. The section at the level of the posterior part of the vomeronasal organ. Abbreviations: *A* anterior, *C* concha of the nasal cavity, *CHG* choanal groove, *CHNG* Choanengang, *D* dorsal, *DNP* nasopharyngeal duct, *DPS* dorsal process of the septomaxilla, *DV* duct of the vomeronasal organ, *EN* external naris, *L* lateral, *M* medial, *MNC* main nasal cavity, *NC* nasal cavity, *OC* oral cavity, *P* posterior, *V* ventral, *VCH* ventral channel, *VNO* vomeronasal organ. Scale bars 100 μm (**f**); 50 μm (**a**, **b**, **d**). 3D reconstructions are not scaled

At the beginning of this stage, the lacrimal duct was visible as a groove in the thicker epithelium on the lateral side of the head, between the anterior part of the eye and maxillary process, at the level of the VNO*.* In the anterior part of this groove a bud was present (Fig. 6a- orange dashed line).

This structure represented the anlage of the posterior part of the lacrimal duct. At the end of this stage, the embryonic lacrimal duct developed considerably and extended from the lateral groove to the Choanengang; and was in close proximity to the entrance of the VNO and anterior to it. The anlage of the lacrimal duct was visible now as a dense area of cells, and there was no evidence of patency in this structure. Its rostral part was in apposition to the Choanengang (Fig. 6b).

#### Stages IV and V

At the beginning of the developmental stage IV, there was still no evidence of a connection of the lacrimal duct and the nasal cavity in any of the embryos that were analysed at that point (not shown).

At the end of this developmental stage (as well as at the stage V), the embryonic lacrimal duct developed considerably and its anterior end was connected to the anterior part of the choanal groove, posterolaterally to the differentiating duct of the VNO (Fig. 6c, d). The embryonic lacrimal duct was filled with cells of various shapes. The wall of the developing lacrimal duct constituted a single layer of columnar cells (see Fig. 6d).

#### Stage VI

The lacrimal duct attained the characteristic tortuosity and its posterior end was slightly dilated (Fig. 6e). Its anterior end reached the dorsal part of the vestigial choanal grove and then the medial wall of the duct of the VNO terminated in a small depression, halfway to the anterior wall of this duct (see Fig. 3a). The lacrimal duct was still non-patent (not shown).

It is worth mentioning that the vestigial part of the choanal groove was entirely outside the forming cupola Jacobsoni. Thus, before reaching the duct of the VNO, the lacrimal duct ran between the remains of the choanal groove and a part of the vomer (see Fig. 6e).

#### Stage VIII

The anterior end of the lacrimal duct extended forward and ran along the medial wall of the duct of the VNO almost reaching the anterior wall of the latter. Behind the duct of the VNO, the lacrimal duct divided and created a finger-like protrusion (the medial diverticulum) that was directed posteromedially (see Fig. 3e).

The tortuosity of the lacrimal duct and dilatation of its posterior end became more pronounced (not shown). It was now wider and, in some restricted areas, patent in its posterior half (Fig. 6f).

#### Stage X

The lacrimal duct became more hollowed and only small areas filled by the cells of various shapes across its length could be distinguished. Although this structure was now patent anteriorly, at the level of the connection with the duct of the VNO, it appeared to still be nonpatent (not shown).

#### Stage XI

**Fig. 7:**
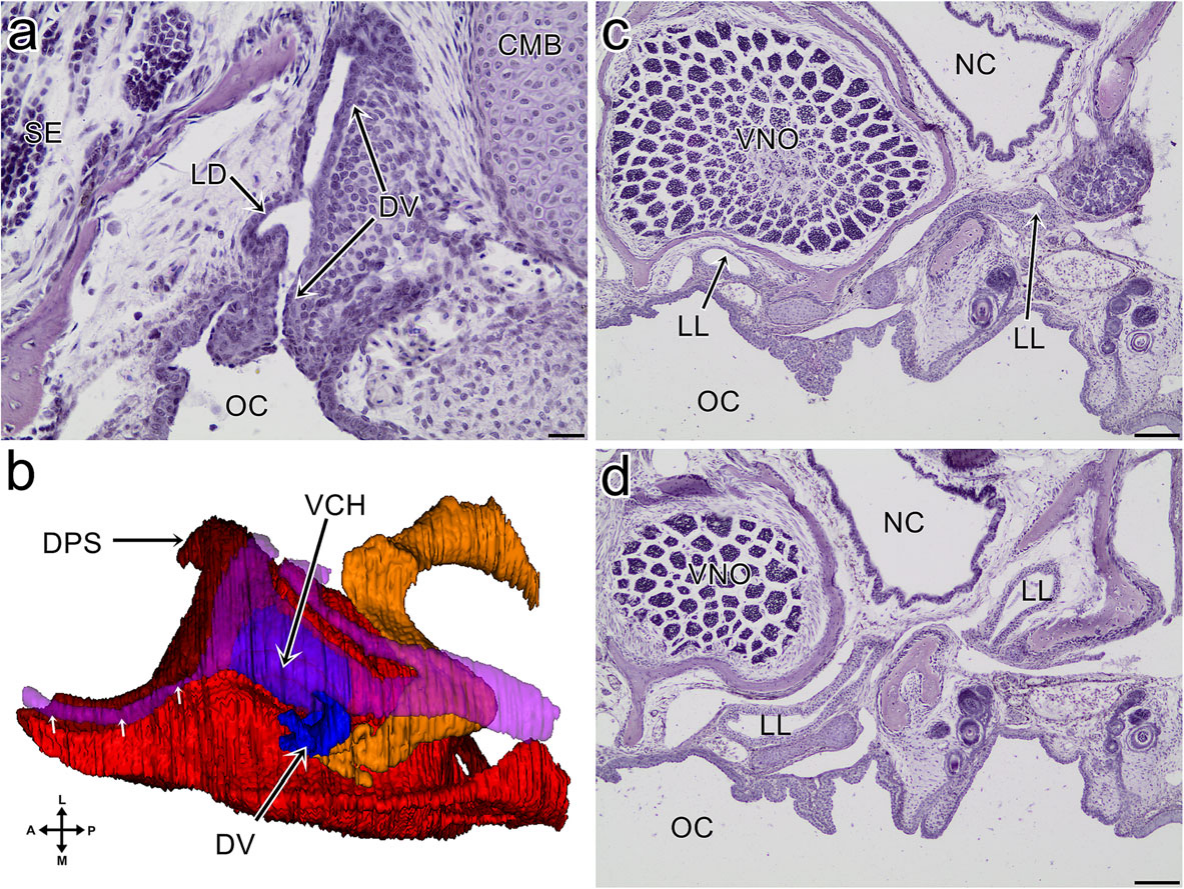
The transverse sections (**a**, **c** and **d**) and 3D reconstruction (**b**) of the lacrimal duct at developmental stage XI. **a** The anterior connection of the lacrimal duct with the duct of the vomeronasal organ. **b** The ventral aspect of the lacrimal duct in relation to the vomeronasal organ, the associated cartilages (*short arrows* indicated the cylindrical part of the lamina transversalis anterior) and the cupola Jacobsoni. The colours of the structures are the same as in Fig. 6e. **c** The larimal duct at the level of the posterior part of the vomeronasal organ. The larimal duct; the section posterior to c. Abbreviations: *A* anterior, *DPS* dorsal process of the septomaxilla, *CMB* cartilage of the mushroom body, *DV* duct of the vomeronasal organ, *L* lateral, *LD* lacrimal duct, *LL* lumen of the lacrimal duct, *M* medial, *NC* nasal cavity, *OC* oral cavity, *P* posterior, *SE* sensory epithelium, *VCH* ventral channel, *VNO* vomeronasal organ. Scale bars 100 μm (**c**, **d**); 20 μm (**a**). 3D reconstruction is not scaled

Because the anteriormost part of the lacrimal duct was patent at this stage, it simply entered into the duct of the VNO from its medial side (Fig. 7a). More posteriorly, it was also possible to distinguish the second area of this entrance, through the anterior part of the lateral diverticulum (see above and Fig. 4b). The tortuosity of the lacrimal duct and dilatation of its orbital end were well developed. Thus, the lacrimal duct probably attained a nearly adult-like form (Fig. 7b). This duct was largely patent (Fig. 7c, d).

### 4. Development of the cupola Jacobsoni and its relation to the vomeronasal nerve

#### Stage II

At this stage, there were no bony or cartilaginous elements in the VNO closure. The indistinctive primordium of the vomeronasal nerve left the anterodorsal part of the developing VNO and reached the anterior telencephalon. At the end of this stage, the vomeronasal nerve became better visible (see Fig. 2b)

#### Stage IV

The elements of the cupola Jacobsoni were not observed yet. The nerve of the VNO was developed and left the dorsal part of the dome of the VNO (not shown).

#### Stage VI

**Fig. 8:**
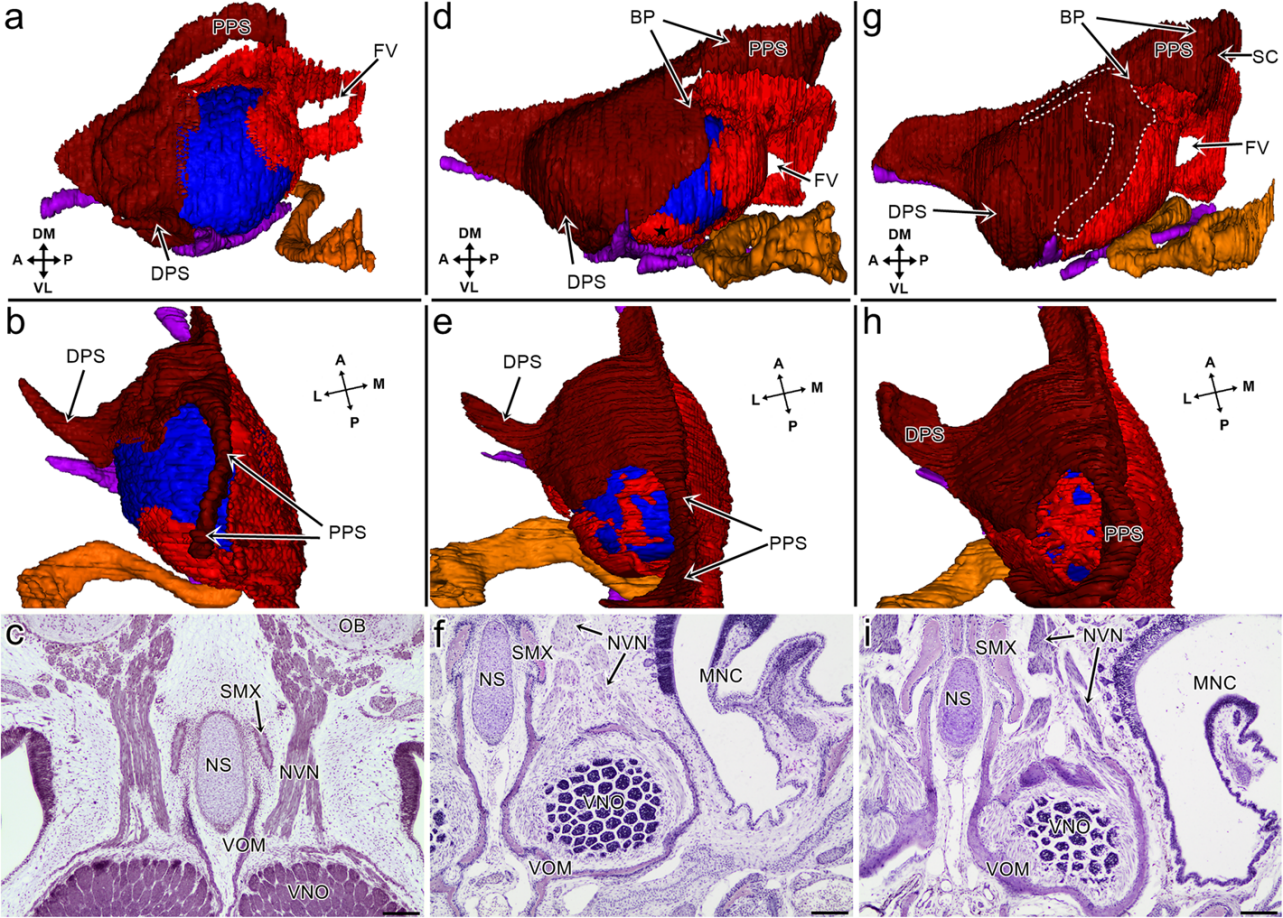
Development of the cupola Jacobsoni and its relation to the vomeronasal nerve in the grass snake. 3D reconstruction (**a**, **b**, **d**, **e**, **g**, **h**) of the cupola Jacobsoni (the colours: the lacrimal duct (*orange*), vomeronasal organ (*blue*), vestige of the choanal groove (*green*), cartilage (*purple*), vomer (*bright red*), and septomaxilla (*dark red*)) and the transverse section at the level of the vomeronasal nerve. **a** The dorsolateral aspect at the end of developmental stage VI. **b** A dorsal view of the bony passage for the vomeronasal nerve at developmental stage VI. **c** Developmental stage VI. **d** The dorsolateral aspect at developmental stage VIII. The star indicates the separate part of the vomer. **e** A dorsal view of the bony passage for the vomeronasal nerve at developmental stage VIII. **f** Developmental stage VIII. **g** The dorsolateral aspect at developmental stage XI. The septomaxillary-vomerine overlapping is indicated by dashed line. **h** A dorsal view of the bony passage for the vomeronasal nerve at developmental stage XI. **i** Developmental stage XI. Abbreviations: *A* anterior, *BP* bony passage for the vomeronasal nerve, *DM* dorsomedial, *DPS* dorsal process of the septomaxilla, *FV* foramen of the vomerine vertical plate, *L* lateral, *M* medial, *MNC* main nasal cavity; *NS* nasal septum, *NVN* vomeronasal nerve, *OB* olfactory bulb, *P* posterior, *PPS* posterior process of the septomaxilla, *SC* septomaxillary condyle *SMX* septomaxilla, *VL* ventrolateral, *VOM* vomer, *VNO* vomeronasal organ. Scale bars 100 μm. 3D reconstructions are not scaled

At the beginning of this stage, elements of the cupola Jacobsoni started appearing. At the end of this stage, this bony closure was well developed (Fig. 8a). It included two bones – the vomer and septomaxilla. The vomer contained very a thin and partially discontinuous (mostly in its central part) vertical lamina, which covered the medial and anteromedial part of the VNO (not shown). The forming cup part extended from the vertical lamina to the ventromedial, posterior and posterodorsal parts of the VNO. Behind the incomplete posterior plate of the vomerine cup, large single foramen was visible on the vertical lamina. The septomaxilla created the anteriormost part of the closure and extended across the dorsal, dorsolateral, lateral and ventrolateral parts of the anterior half of the VNO. At that time, two processes were observed. The dorsal process extended from the lateral part of the forming septomaxillary plate. The second, posterior process (the future posterodorsal part of the septomaxillary plate and septomaxillary condyle) protruded from the dorsal part of the plate (see Fig. 8a).

The posterior process of the septomaxilla emerged above the VNO and the vertical plate of the vomer (see Fig. 8a). There was no contact between this septomaxillary process and the vomer. Moreover, laterally from this area, the VNO was open dorsally to a significant extent, and there was no significant barrier for the route of the vomeronasal nerve (Fig. 8b, c).

The large gap in the bony closure continued laterally and then ventrally. The duct of the VNO, and the majority of the lateral part of the ventral channel, remained uncovered by bones (see Fig. 6e)

Within this partial closure, the vomer and septomaxilla laid in apposition anteriorly, and left only the anteromedial slit-like gap between them, which was partially filled by the anterior half of the lamina transversalis anterior. This cartilage was visible there in the form of a cylindrical structure (see Fig. 6e short arrows). This cartilage ran further anteriorly to the connection with the nasal septum; however, before leaving the cupola Jacobsoni, it created a short dorsal projection that also filled the anteroventral part of the apposition of the vomer and the septomaxilla (not shown). The posterior half of the lamina transversalis anterior, which was significantly wider than the anterior part, was located just under the ventral gap in the bony closure of the VNO. Thus, the ventral space for the duct of the VNO was restricted by this cartilage and the vomer (see Fig. 6e). The posterolateral, and most of the lateral part of the ventral channel, ran in the cartilaginous groove that had been created by the base of the mushroom body cartilage and the rest of the adjacent part of the lamina transversalis anterior (see Figs. 3d and 6e)

#### Stage VIII

The cupola Jacobsoni was now more complete due to the development of the posterior border of the septomaxilla and vomerine cup in the posterior part of the VNO. The anteriormost part of the septomaxilla formed a well visible rod-like structure. The dorsal process became more solid and was directed more vertically. A separate part of the vomer was visible on a small area of the ventrolateral part of the VNO (Fig. 8d).

The vertical lamina of the vomer became thicker. Posteriorly, it was developed dorsally and reached the posterior process of the septomaxilla. The latter extended more posteriorly, its end turned laterally and formed the anlage of the septomaxillary condyle. The posterodorsal part of the forming vomerine cup created a raised area that was directed dorsally toward the anlage of this condyle, although it did not reach the latter. All of these changes created the medial, posteromedial and incomplete posterior wall of the bony passage for the vomeronasal nerve. The bony passage was restricted anteriorly and anteriolaterally by the slightly raised area of the septomaxilla. It remained open laterally and posterolaterally (Fig. 8e and see Fig. 8d). Additionally, the vomerine bridges were present on the bottom of the bony passage. Thus, the access of the vomeronasal nerve to the VNO was restricted and was possible now through two foramina (larger medial and minor lateral) and space between the vomerine bridges and the septomaxilla (Fig. 8f and see Fig. 8e).

The lamina transversalis anterior and the hypochoanal cartilage were connected posteriorly through the ectochoanal cartilage (see Fig. 5f).

#### Stages X and XI

According to the obtained material, it appears that the cupola Jacobsoni was very similar in these two stages. The VNO was largely enclosed in the cupola Jacobsoni (Fig. 8g). The septomaxillary condyle attained its characteristic hook-like shape and expanded ventrally, almost reaching the vomerine cup. The rod-like structure and the dorsal process of the septomaxilla became more massive. The posterior border of the septomaxillary plate developed posteriorly and, in the region of the anterior border of the bony passage, a raised flange, which overlapped the vomer, was formed. On the lateral side of this structure, a process directed posteriorly toward the end of the hooked condyle could be observed. Thus, the bony passage for the vomeronasal nerve remained open laterally (Fig. 8h and see Fig. 8g). At the bottom of the bony passage, the vomer created multiple foramina for the vomeronasal nerve (Fig. 8i and see Fig. 8h). Anteriorly to this, where the vomer was overlapped by the septomaxilla, the cupola Jacobsoni appeared to be incomplete because of the presence of an indentation rather than an openings. The septomaxillary-vomerine overlapping continued downward on the lateral wall of the cupola Jacobsoni (see Fig. 8g- dashed line). Thus, at this stage, the vomer was well developed on the lateral wall of the cupola Jacobsoni and there was no separate part of this bone (see Fig. 8g).

The ventral covering was more complete – primarily due to the development of the vomer. A large hole, the fenestra vomeronasalis externa, now appeared for the duct of the VNO and for most of the lateral part of the ventral channel. The septomaxilla separated this opening anteriolaterally. The rest of the borders were created by the vomer. The ventral space for the route of the VNO duct was more restricted by the lamina transversalis anterior and the vomer (see Fig. 7b).

### 5. Specialisation of the vomeronasal sensory epithelium

#### Stage II

**Fig. 9:**
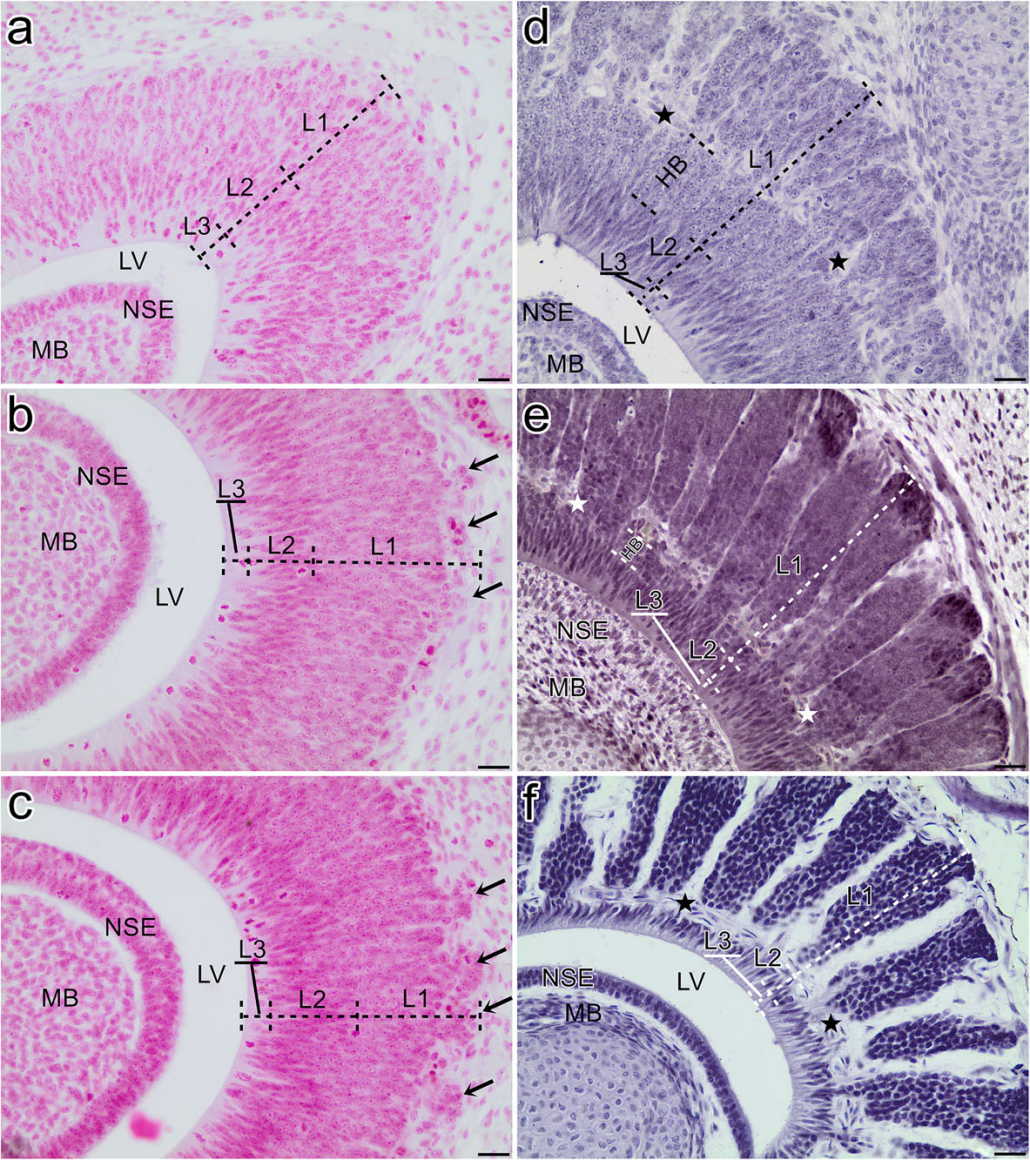
Development of the sensory epithelium of the vomeronasal organ in grass snake. **a** The beginning of developmental stage II. **b** The end of developmental stage II. The rounded projections to the surrounding mesenchymal tissue are visible (*short black arrows*). **c** The beginning of developmental stage IV. The rounded projections (*short black arrows*) are well visible. **d** The end of developmental stage IV. **e** The end of developmental stage VI. **f** Developmental stage XI. *Stars* indicate the level of extension of columns within the basal layer (**d**, **e**, **f**). Abbreviations: *HB* homogenous part of the basal layer, *LV* lumen of the vomeronasal organ, *L1* basal layer, *L2* central layer, *L3* peripheral layer, *MB* mushroom body, *NSE* non-sensory epithelium. Scale bars 100 μm

The sensory epithelium of the dorsal dome was considerably thicker than the non-sensory epithelium of the mushroom body (Fig. 9a). The transverse section of the sensory epithelium reached its maximum thickness on the dorsal side of the organ and decreased gradually in thickness towards the transition zone between the sensory and non-sensory epithelium. Three layers were distinguishable within the dorsal dome (see Fig. 9a). The basal layer (L1) included cells that had circular, oval or elongated nuclei and constituted the thickest part of the sensory epithelium. A slightly thinner, central layer (L2) was filled with cells that had elongated and more darkly stained nuclei. The thinnest, peripheral layer (L3) that faced the lumen consisted of few nuclei. Many mitotic figures were present in this layer. The lumen surface of the sensory epithelium was covered by the developing brush border membrane. The non-sensory epithelium consisted of two or more layers of undifferentiated cells.

At the end of this developmental stage, the basal surface of the sensory epithelium became irregular due to the presence of rounded projections into the surrounding mesenchymal tissue (Fig. 9b- short black arrows).

#### Stage IV

At the beginning of the developmental stage IV, the sensory and non-sensory epithelia of the VNO primordium were almost the same as at the previously described stage. Two changes occurred within the basal layer (L1) of the sensory epithelium. It now contained only the cells that had circular or oval nuclei. The external rounded projections of the basal layer into the mesenchymal tissue became well visible (Fig. 9c- short black arrows). In the slightly older embryos at this stage, the differentiating mesenchymal cells divided the basal layer of the sensory epithelium of the VNO into agglomerations that had spherical shapes (not shown). The developing brush border membrane also became more distinctive. At the end of this period, the agglomerations became elongated, and thus, adopted a columnar shape (Fig. 9d). In the thickest part of the dorsal dome, these structures occupied more than 50% of the height of the basal layer (L1) of the sensory epithelium. The compartments of the mesenchymal cells were relatively narrow and therefore, columns were not well distinguished. Single blood cells were also visible at the top and base of these compartments. On the histological sections, the columns were not completely connected with the remaining sensory epithelium because differentiating mesenchymal cells were visible at the apical part of the developing columns. The sensory epithelium of the basal layer outside the differentiating columns were visible as a homogenous layer (see Fig. 9d- HB). The central (L2) and peripheral layer (L3) exhibited the same pattern as at the developmental stage II.

#### Stage V

The developing columns of the sensory epithelium were of the same height as at the end of the previous stage. The borders between these agglomerations became more distinctive, but the compartments of the developing connective tissue were still very narrow. There were no other significant changes (not shown).

#### Stage VI

At the developmental stage VI, the sensory epithelium of the VNO of the grass snake developed considerably. During this period, the epithelial columns of the basal layer (L1) became higher. At the end of this stage, these structures extended through almost the entire thickness of the basal layer (L1) and left only a few rows (about 2–3) of the cells (HB) facing the central layer layer (L2). Moreover, the borders between the columns were clearly visible (Fig. 9e). All of these were the results of the expansion of the compartments that had been created by the developing connective tissue. In the cross section, columns were polygonal or rounded (see Fig. 3b). The central (L2) layer with cells, that were more darkly stained and had elongated nuclei, became thinner and, finally, contained only about 2–3 rows of cells. At the end of this developmental stage, the cells of the basal layer (L1) and those from the central layer (L2) could not be distinguished based on the staining character. In some apical and basal parts of the developing connective tissue, which occurred between the columns, in addition to the single blood cells, the walls of the blood vessels were distinguishable. Within the peripheral layer (L3) that faced the lumen, some fibrous structures that were nearly perpendicular to the surface of the VNO lumen were visible. The mitotic figures were still present there, but they were now very rare (see Fig. 9e).

In addition to the lateral and posterolateral parts of the spiral channel, it constituted a transition zone between the sensory and non-sensory epithelium. The most distinctive and ventrally developed part of this structure was lined with the non-sensory epithelium (see Fig. 3d).

#### Stage VIII

The spaces between the columns of the sensory epithelium were well developed on the histological sections. These structures extended through the entire thickness of the basal layer and thus, the undifferentiated homogenous part of this layer disappeared. Therefore, at this stage, the “connection zones” across the connective tissue occurred directly between the cells (that had circular or oval nuclei) of the basal layer and the cells (that had elongated nuclei) of the central layer. Additionally, the cells of the spindle-like nuclei could be distinguished in the walls of the columns. The clear mitotic figures were not observed within the peripheral layer from now on (not shown).

#### Stages X and XI

In comparison to the developmental stage VIII, the central layer (L2) of the cells that contained elongated nuclei was reduced to a single row of cells. The fibrous structures of the peripheral layer (L3) and the brush border membrane were now clearly visible (Fig. 9f).

**Fig. 10:**
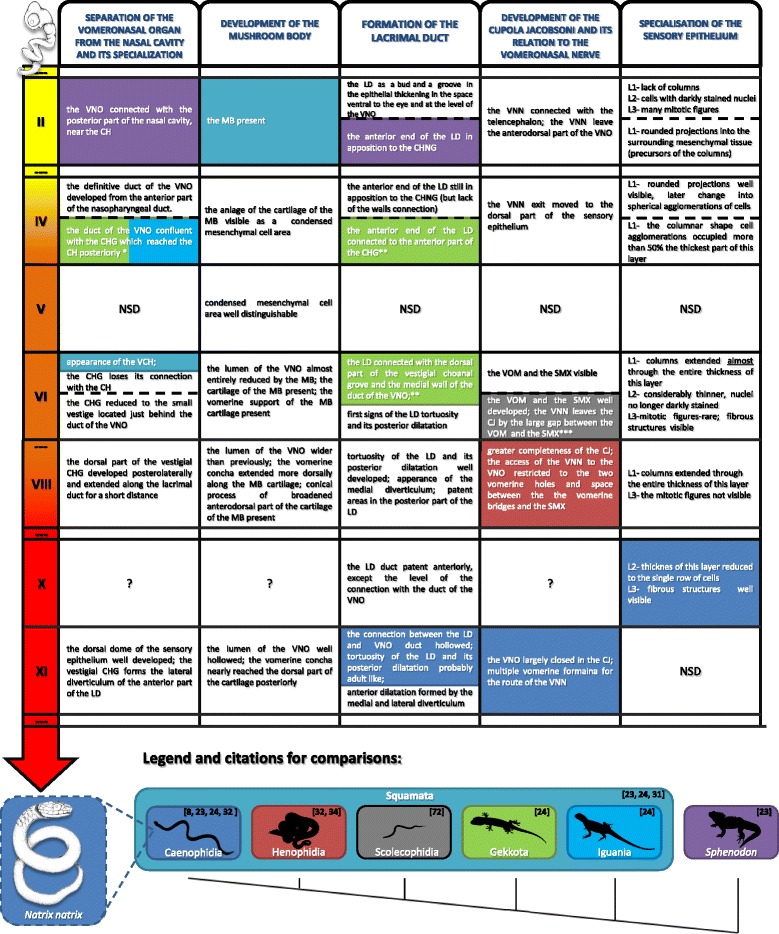
Summary of the results of this study and comparisons to adult structures of other squamates and *Sphenodon*. *This condition occurs in both, Gekkota and Iguania [24]. ** In Gekkota the lacrimal duct is connected to the choanal groove and the medial wall of the duct of the vomeronasal organ simultaneously. Moreover, the choanal groove in adults geckoes is not vestigial [24]. *** In some species of Scolecophidia the vomeronasal nerve enters the organ through a vertical foramen in the septomaxilla [42, 43]. Silhouettes in the legend (except Henophidia) from http://phylopic.org/. Silhouettes of the grass snake embryo in the timeline from Rupik [59]. In some cases the beginning and the end of the stage are separated by the dashed line. Abbreviations: *CH* choana, *CHG* choanal groove, *CHNG* Choanengang, *CJ* cupola Jacobsoni, *MB* mushroom body, *LD* lacrimal duct, *L1* basal layer, *L2* central layer, *L3* peripheral layer, *NSD* no significant differences, *SMX* septomaxilla, *VCH* ventral channel, *VOM* vomer, *VNN* vomeronasal nerve, *VNO* vomeronasal nerve

The main developmental changes were summarized and defined as certain levels of anatomical or histological complexity in Fig. 10.

## Discussion

### Separation of the VNO from the nasal cavity and its specialization

The VNO in terapods exhibits great variations with respect to the association with the nasal cavity [22, 23, 36, 63]. In living amphibians, the VNO is a diverticulum of the nasal cavity [4, 64]; in turtles only the vomeronasal epithelium, which covers certain part of the main nasal cavity, can be distinguished [65]; in tuatara the VNO is a lens-shaped structure that lies along the nasal septum and is connected to the anterior part of the nasal cavity [37, 66], while in squamates it forms a well-developed structure that has a direct connection with the oral cavity and an indirect connection with the nasal cavity may occur only through the choanal groove in some lizards [24, 25, 31]. The VNO in mammals lies along the nasal septum and in adults may be connected directly to the nasal cavity [67, 68] or to both, the nasal and oral cavity [21]. It is worth mentioning that the VNO is absent in most fishes [1, 3, 69], adult crocodilians and birds [22, 23, 63, 70], while in some bats, chimpanzees and humans only a “nonchemosensory vestige” is present [18].

In Squamata, the primordium of the VNO arises from the nasal pit where it initially creates the ventromedial groove invaginated into the frontonasal mass, [22, 23, 28, 29].

The separation of the VNO from the nasal cavity occurred very early. We were not able to access the earliest stages of embryogenesis, but it appears that this process is conservative in the squamates evolution and that it starts as a narrowing of the connection with the nasal sac [23, 28]. After the fusion of the medial and lateral nasal prominences, the localisation of the connection moved gradually from the level of the Choanengang to the choana one. This pattern is, generally, in accordance with the descriptions of Parsons [23]. However, some differences could occur in order to identify some structures, due to the lack of a definitive border between the Choanengang and the nasopharyngeal duct and between the choana and the choanal groove. In some groups of lizards, the connection between the nasal cavity and the VNO may occur only through the palatal choanal groove, which constitutes the remnants of the primitive choana [24, 31]. Our study shows the development of this structure in the grass snake embryos and sheds light on the presumptive presence of this structure in adults snakes. Bellairs and Boyd [24] stated that the choanal grooves are generally absent in snakes, but in taxa such as *Natrix*, which are not characterised by a shortening of the maxilla, a superficial palate exhibits a depression in the area “between the anterior part of the maxilla and the septomaxilla.” However, they did not mention whether this structure could be a homologue to the choanal groove or could be functional in the context of the delivery of chemicals to the VNO (see below). Additionally, the exact location of this depression on the palate is difficult to identify. McDowell [26] suggested that the groove flanking the structure called the “vomerine raphe” seems to be homologous to the choanal groove. The “vomerine raphe” is a narrow median ridge on the anterior part of the palate. He indicated that the mentioned flanking area constitutes “a natural mould of the tines of the tongue” and contains the fenestra vomeronasalis externa (see Fig. 1b- white arrow). According to him, the latter supports the view about the homology. In contrast to these statements, our study shows that the choanal groove disappears entirely during embryogenesis in the grass snake. Stage VI is crucial in this process. At the beginning of this stage, the choanal groove is present but does not reach the choana. Later, only a small vestige of it remains just posterior to the duct of the VNO. Before hatching, this vestige becomes the presumptive lateral diverticulum of the anterior part of the lacrimal duct. McDowell’s view could have been affected by his considering only the surface of the palate of adult animals. Thus, in adult grass snakes, the choanal groove is absent and this is probably a synapomorphy of snakes. In fact, the morphology of the choanal groove and its relation to the duct of VNO were used to reconstruct the phylogeny of Squamata [71]. As we have mentioned, the embryonic choanal groove of the grass snake was not supported by a cartilage or bone, but in adult lizards this structure is supported by an ectochoanal cartilage extending from the lamina transversalis anterior [72].

In adult lizards (“non-ophidian squamates”), the anterior extent of the choanal groove exhibits some variation. It can be directly continuous with the duct of the VNO (Iguania and Gekkota), can be separated from the duct of the VNO and end anteriorly at a distance behind this duct (is relatively long in anguids, amphisbaenids and Xantusiidae; and short in teiids; and very short in monitor lizards), or it can terminate behind the duct of the VNO, but reach it by the anteriormost part of the lacrimal duct, which, with the choanal groove, forms the structure called lachrymo-choanal gutter (skinks and lacertids) [24]. Finally, it can be entirely absent, at least in one species of Dibamidae, *Dibamus taylory* [73].

Pratt [31] suggested that, in the taxa in which the choanal groove is well developed, the particles that are delivered to the oral cavity by the tongue are then carried to the VNO by the cilia of this groove and/or the surface of the mushroom body. In this supposed mechanism, the stream of lacrimal fluid that passes through the choanal groove to the duct of the VNO, or its vicinity, plays a crucial role. In the taxa in which the choanal groove is reduced or absent, as in monitor lizards or snakes, this view must be rejected.

At the time when the choanal groove became significantly reduced (developmental stage VI), substantial changes in the general shape of the VNO, including the shape of the duct and the appearance of the ventral channel, were observed. Pratt’s “ciliary hypothesis” [31] assumes that the spiral channel is involved in the drainage of the VNO lumen. This structure is probably homologous to the ventral channel described here. The most widely accepted hypothesis for the mechanism for the movement of chemicals through the vomeronasal fenestrae and into the lumen of the VNO is suction [25, 39, 49, 51] (see discussion about the mushroom body). Thus, it seems possible that no special drainage system of the VNO lumen is required. However, our observations may support some proposal of Pratt [31]. The proposal of the drainage function of this part of the spiral channel is supported by three observations that were found in this structure in our study. First, it is lined by the non-sensory epithelium, and thus, is not directly involved in chemoreception; second, it is sloped ventrally towards the duct of the VNO, which may improve the fluid outflow from the lumen into the oral cavity; and third, presence of the cartilage groove for the lateral part of this channel and its ventrolateral position in relation to the mushroom body which may prevent the VNO lumen from being reduced during the elevation of the floor of the mouth (see the discussion about mushroom body). Moreover, Young [51] indicated that the “ciliary hypothesis” would require separation of two flows (the “new” portion of the particles moving dorsally into the duct and the “old” portion of the particles moving ventrally from the lumen of the VNO into the oral cavity), and that the duct of the VNO would be constantly open, while “there is no anatomical specialization”. However, our study shows that the duct of the VNO is not a simple and extremely narrow tube, but it can adopt a flattened tube shape with a cylindrical eminence on the lateral side. Unfortunately, it appears that the oldest embryos exhibit a duct of the VNO which is not entirely hollowed, and therefore, we were not able to access the adult-like shape of this lumen.

The ciliary currents have been considered to be playing a secondary role in chemical delivery – as opposed to suction, which involves the elastic recoil of the mushroom body cartilage [60]. Our observations may support this view and Pratt’s [31] hypothesis may also be true for snakes. However, lack of the choanal groove in this clade requires some other mechanism of transferring chemical molecules from the tongue to the vomeronasal opening.

### Development of the mushroom body

As mentioned before, the most widely accepted hypothesis for the mechanism of the movement of chemicals through the vomeronasal fenestrae and into the lumen of the VNO is suction, which is associated with the mushroom body [25, 39, 49, 51]. The concha of the VNO is absent in *Sphenodon*, thus its presence seems to be a squamate apomorphy [22, 23, 71]. However, this structure is either poorly developed or absent in Iguania [31]. A well distinguishable mushroom body primordium of the grass snake was present at the beginning of stage II. Thus, it appears, probably, in the embryos of *N. natrix* before the eggs are laid. In *Thamnophis*, this structure appears soon after the fusion of the medial and lateral nasal processes [23]. The same situation exists probably in *Malpolon monspessulana* [28]. The cartilage of the mushroom body appears later, at the beginning of stage VI. It is connected ventrally with the remaining part of the lamina transversalis anterior. The broad part of the lamina transversalis anterior, which directly gives the dorsal projections of the cartilage of the mushroom body and which also passes posteriorly into the ectochoanal cartilage, has been often called the cartilage of Jacobson’s organ [35, 72]. It can form the sole part of the lamina transversalis anterior (in scolecophidians and some viperids) or constitute the most developed part of this cartilage– in comparison to the separate, vestigial anterior one (elapids and some viperids) [72]. At the same time when the cartilage of the mushroom body appears, the basal part of the mushroom body cartilage gains the anteromedial bony supports from the vomer. The latter develops considerably at later stages. The vomerine concha was also found in monitor lizards [24, 38].

### Formation of the lacrimal duct

The posterior connection of the lacrimal duct with the conjunctival or sub-brillar space, where the orbital glands discharge, is evident in Tetrapods (e.g. [13–15, 40, 74]). Secretion of the primary orbital gland, the Harderian gland, was traditionally thought to be linked with the lubricative function in the eye, but in various taxa numerous functions at the anterior end of the lacrimal duct have been ascribed to this gland, including vomeronasal sense, immune response, pheromone production and thermoregulation [13]. A presumed role in vomerolfaction has been suggested especially for squamate reptiles in which a relatively close association occurs between the lacrimal duct and the Harderian gland and the lacrimal duct and the duct of the VNO or choanal groove [23, 40, 41]. However, also in other taxa, in which this association is not as evident, as in mammals, anurans, urodeles and *Sphenodon*, a functional interaction between the Harderian gland and the VNO may be present [15, 74].

In “typical” lizards, the lacrimal duct opens to the conjunctival or sub-brillar space through two canaliculi anterior to the Harderian gland, and is separated from it by the nictitating membrane in these taxa in which it is present. In snakes, this connection is more intimate – especially in colubrids in which the Harderian glands is confluent with the lacrimal duct. But in more primitive conditions, in boids (Henophidia) and Scolecophidia, the Harderian glad discharges to both the sub-brillar space and the lacrimal duct [40, 57]. Interestingly, these two snake conditions are similar to these present in pygopodids and amphisbaenids, respectively [40, 41].

The final destination of the Harderian gland secretion, drained by the lacrimal duct, varies among Squamata. The anterior end of the lacrimal duct of snakes, monitor lizards pygopodids, some amphisbaenids, and at least one dibamid (*Dibamus taylory*), reaches the VNO duct, and there is no other communication with the oral cavity posteriorly to this level [24, 33, 57, 73]. A similar situation occurs in Xantusiidae. However, in this case, the lacrimal duct discharges in the lumen of the VNO: “just dorsal and medial to the origin of the duct of that organ” [24]. In the remaining members of the squamate reptiles, communication (direct or indirect) with the superficial palate exhibits some variation and is associated with the variation of the choanal groove (see above). Generally, in lizards, the lacrimal duct communicates with the choanal groove by a more or less extensive posterior opening (or openings) and, finally, opens into the VNO duct (gekkotans, teiids) or into adjacent region of the superficial palate (anguids), or into both (lacertids, skinks and some amphisbaenids). The lacrimal duct and the choanal groove appear to be especially closely associated in skinks, lacertids and chameleons, in which the two structures form the “lachrymo-choanal gutter,” and the border between these two components is difficult to determine. However, the posterior communication of the lacrimal duct with the choanal groove in the rest iguanians and in anguids is also extensive [24, 38].

The lacrimal duct of *Sphenodon* discharges into the anterior part of the nasal cavity near the choana and at some distance behind the opening of the VNO duct [13, 23, 24, 37]. This may represent the ancestral condition for Squamata.

Our study showed that, during the embryogenesis of the grass snake, the lacrimal duct “follows” the duct of the VNO and, finally, enters the medial wall of the duct of the VNO organ. Thus, different conditions, in relation to the connection of the anterior end of the lacrimal duct with the nasal cavity and the superficial palate (the choanal groove), can be distinguished. These different conditions may resemble these present in some adults lizards. Parsons [23] found that, in *Thamnopsis*, the lacrimal duct, initially, enters the ventromedial part of the nasal cavity, i.e. the Choanengang. In later embryos, the duct moves gradually toward the duct of the VNO and its rostral end receives the choana, the palate between the choana and the duct of the VNO, the palate at the posterior end of the duct of the vomeronasal and, finally, the ventral end of the duct of the VNO. Our description differs from this. We did not find the state in which the lacrimal duct entered directly into the palate. However, our observations suggest that at the developmental stage VI, this structure is associated with both the medial wall of the VNO duct and the vestigial choanal groove. The latter has never been described. Its position in relation to the duct of the VNO and its general shape suggest that this interpretation is correct. Considering the vestigial choanal groove as a part of the lacrimal duct, confirms Parsons’ view [23] (apart from the fact that the anterior part of the lacrimal duct is probably not patent at this time). Lack of any information in his description about the connection of the lacrimal duct at the same stage with the duct of the VNO may be a result of using slightly younger (with respect to developmental stage VI of the grass snake) embryos in his analysis. Another interpretation might be that the residue of the choanal groove could be considered to be the posterior part of the duct of the VNO. In fact, both structures (the choanal groove and the duct of the VNO) have been recognised as the remnants of a primitive slit-like choana [24]. Our observation also supports this view. However, at the end of embryogenesis, the residual choanal groove loses its connection to the palate and becomes a part of the lacrimal duct (the lateral diverticulum). Thus, it is reasonable not to consider this remnant of the choanal groove as the VNO duct. Nevertheless, it was difficult to access the patency of the connection between the presumptive lateral diverticulum of the lacrimal duct and the rostral end of the “original” lacrimal duct. Thus the study of adult grass snake anatomy is required.

Bellairs and Boyd [40] proposed that just like the chemical particles from the tongue, the Harderian secretion may be sucked from the lacrimal duct into the lumen of the VNO and act as an “additional solvent.” In fact, secretion of the Harderian gland was found inside the lumen of the VNO and on the surface of the tongue [13, 14]. Moreover, it was shown that the female pheromones of *Thamnophis*, insoluble in an aqueous solution, became soluble after adding to them the Harderian gland homogenate [16]. Close anatomical association of the anterior end with the VNO duct may result in snakes in a direct delivery of the “additional solvent” into the VNO and the tines of the tongue, and thus, facilitate the delivery, of the chemical particles to the lumen of the VNO. In many lizards, the secretion of the Harderian gland must be delivered into the choanal groove. Thus, this delivery seems to be dispersed, and most of the fluid can reach the dorsal surface of the tongue.

Characteristic dilatation of the posterior part of the lacrimal duct in snakes was considered to be a reservoir of the fluid of Harderian gland [40]. Our research showed that the anterior part of the lacrimal duct is characterised by the medial and lateral diverticuli that are created by the original growth of the lacrimal duct and the vestige of the choanal groove, respectively. The precise role of these structures remains unknown, but we suspect that these two diverticula may drain any excess of the Harderian secretion, thus perhaps, consitute the anterior reservoir.

The posterior dilatation of the lacrimal duct developed in parallel with the increase of the level of its tortuosity. The latter is probably associated with the rate of the proliferation in the anterior part of the lacrimal duct which exceed the linear growth of the relatively short snouts of snakes [24]. Souza et al. [27] divided analysed snakes (colubrids and boids) into three groups according to the course of the lacrimal duct – from the least to the most extreme tortuosity. However, both boids and colubrids were placed into the first two groups, thus it is impossible to define the plesiomorphic or apomorphic state for snakes. Thus, the route of the lacrimal duct in snake may exhibit great evolutionary plasticity.

### Development of the cupola Jacobsoni and its relation to the vomeronasal nerve

Two bones, the vomer and the septomaxilla, were considered in the present study as connected with the VNO. The exclusion of the maxilla from the lateral wall of the cupola Jacobsoni is considered to be a synapomorphy of snakes [32]. The vomer and septomaxilla, generally closely fitting to each other in adults snakes, are intramembranous bones and develop as an independent condensation [29]. Interestingly, our observation showed, that, at least in the grass snake and probably in other colubrids, the small plate, separated from the main mass of the vomer, is present on the ventrolateral part of the VNO at the developmental stage VIII. At later stages this plate became indistinguishable due to its fusion with the remaining part of the vomer.

A comparison between the craniofacial development in different snakes species indicates that the vomer and septomaxilla start to ossify at the same time or that the vomer appears shortly before the septomaxilla [29, 75, 76]. In our specimens, these two bones were observed for the first time at the beginning of the developmental stage VI and were well developed at the end of this stage.

Unlike mammals, the vomer constitutes a significant support for the roof of the oral cavity in Diapsida, excluding crocodilians [77]. However, within the squamate reptiles the horizontal posterior lamina is generally absent or reduced, as in Caenophidia [34]. In lizards and snakes, the vomer is not only a part of the external covering of the VNO, but it also provides a structural support for the mushroom body cartilage by means of the process called vomerine concha [24, 38].

The septomaxilla is absent in birds, crocodilians, turtles and the majority of the extant mammals. In snakes and lizards (except chameleons), it is a part of the bony closure of the VNO, forms the floor of the nasal capsule and its medial border is closely apposed to the nasal septum [32, 77–79]. Lack of the septomaxilla in chameleons seems to be associated with reduction or absence of the VNO [72]. In anurans and urodeles, the septomaxilla is connected with the route of the lacrimal duct [78]. The same has been also suggested for non-mammalian synapsids [79]. It is not the case in lizards and snakes and even in *Sphenodon* [78]. These suggest that the septomaxilla’s loss of the close connection with the lacrimal duct took place at least in the ancestor of the squamate reptiles. We have not found any close spatial relationship between the route of the lacrimal duct and the septomaxilla. In fact, the former was located outside the forming bony closure of the VNO. We found that, in the grass snake embryos, the lacrimal duct is connected to the medial wall of the VNO duct at least since the developmental stage VI, when the septomaxilla occupied a rather anterior part of the forming VNO bony closure. Additionally, at later stages, the posterior part of the septomaxilla is located dorsally to the anterior end of the lacrimal duct and at significant distance from it.

The hook-shaped septomaxillary condyles, located on the posterior end of these paired bones, form a joint surface for the ventral edges of the frontal bones [35]. We found that each condyle, together with the rest of the posterodorsal part of the septomaxilla, constitutes a bony passage for the route of the vomeronasal nerve. This kind of incomplete bony tube has never been described in this context.

In lizards, the completion of the cupola Jacobsoni exhibits a great deal of variation. The bony closure of the VNO in Autarchoglossa is generally more developed than in gekkotans. In iguanians, the cupola Jacobsoni is considered to be absent due to the presence of only a small and flat septomaxilla and lack of established contact between the vomer or septomaxilla with the maxillary lingual shelf [32]. The autarchoglossan vomeronasal nerve reaches the VNO through the more or less restricted posteromedial gap between the vomer and septomaxilla and runs, at least partially, through the supravomerine canal [32, 38]. However, the modified autarchoglossan condition occurs in advanced snakes (Caenophidia), and at least in one dibamid (*Dibamus novaeguineae*). The bony closure in these taxa is complete, and thus, the vomeronasal nerve never pierces the vomer [32, 34]. Consequently, presence of the multiple well-developed vomerine openings, characteristic for caenophidians, observed in our study at the last analysed stage, provides dorsal pathways for the VNO nerve. At the earlier stage (VIII), the vomer creates bridges that restrict access to the organ for the vomeronasal nerve to two relatively large holes and a space between the vomer and septomaxilla. This resembles the condition characteristic of the more basal snakes, Henophidia, which are characterized by one, two or rarely three openings for the vomeronasal nerve [32, 34]. Interestingly, in scolecophidians which are the most basal extant snakes, the connection of the vomeronasal nerve with the VNO is restricted to the posterior gap between the vomer and septomaxilla (condition similar to this occurring in lizard-like autarchoglossans), or the vomeronasal nerve enters the organ through a vertical foramen in the septomaxilla [42, 43, 72, 80].

The condition of the cupola Jacobsoni that was observed in the oldest analysed embryos appeared to be incomplete. This was indicated by an indentation rather than presence of openings anterior to the bottom of the bony passage. Thus, some changes probably occur during postembryonic life.

Despite the fact that the term “cupola Jacobsoni” is generally limited to the bony elements covering the VNO [32], the lamina transversalis anterior seems to constitute an important component of this closure [33–35]. Indeed, in lizards, in which the bony closure is generally less complete, the cartilaginous covering is significant and contains not only the lamina transversalis anterior, but also the nasal septum and paraseptal cartilage [75]. Our visualizations showed that, in the grass snake embryos, the anterior part of the lamina transversalis anterior filled the ventral and anteroventral part of the anteromedial slit-like gap between the vomer and septomaxilla. This thinner part of the lamina transversalis anterior probably allows for movability in the septomaxillary-vomerine complexes, which influences movements of the VNO duct. This may be associated with its chemosensory function [35].

The primordium of the vomeronasal nerve was present in the youngest embryos analysed in this study and it had already reached the anterior telencephalon. In Squamata, the nerve fibres from the vomeronasal epithelium do not mingle with those from the olfactory epithelium [22, 23, 63, 81]. It was also found that the olfactory nerve reached the brain structures before the vomeronasal nerve [28, 55].

### Specialisation of the vomeronasal sensory epithelium

One of the most characteristic features of the sensory epithelium of the VNO in snakes is presence of the columnar compartments containing bipolar neurons (e.g. [8, 19, 23, 44]). There is still not much information about the morphological diversity of this structure in non-ophidian squamates. The role of the capillary network which surrounds the columns of the VNO sensory epithelium, has been considered in the context of the metabolic needs of neurons [55], or as a structure that is involved in the mechanism of the delivery of chemicals to the VNO [24]. If the latter is true, then the sensory epithelium of the VNO may be another structure involved in changing the volume of the VNO. It has been proposed that the contraction and relaxation in the capillary network around and within the sensory epithelium may be involved in this process [24, 82]. A similar mechanism occurs in mammals in which the vascular plexus that surrounds the vomeronasal lumen may act as a pump [21, 83]. However, because of lack of a “mammalian vascular pump” in squamate reptiles, this explanation can only be applied to the snakes and lizards that have the sensory epithelium columns that are separated by connective tissue with blood vesicles.

The process of the columns formation in snakes and other Squamata remains poorly understood. On the basis of electron microscopic studies in adult squamate reptiles [8, 19, 44, 45], we propose the following identification of three layers. The basal layer (L1) represents the precursors of the bipolar neurons, the central layer (L2) contains the supporting cells, and finally, the peripheral layer (L3), facing the lumen of the VNO lumen, appears to constitute a layer of the dendrites and protrusions of the supporting cells. The cells with the spindle-like nuclei, which were located in the walls of the columns at least from stage VIII, may represent the satellite cells.

The columns of the sensory epithelium probably start forming at the end of stage II as an irregular surface of the basal layer. When the columns appear at stage IV, the basal layer is well subdivided into basally located columns and an apical part without these structures. The part of the basal layer that was undifferentiated into columns appears to be the “intermediate receptor layer” that was described by EL-Din and Dakrory [28]. This layer was not distinguished in the *Thamnophis* embryos [55, 56]. Thus, the entire part of the sensory epithelium under the forming columns was considered to be a supporting cells layer. However, in our material we did not find any evidence of the homogeneity of this part of the sensory epithelium, and, according to the nucleus shape and the staining character, two populations of cells were visible even at the earliest analysed stage. Thus, it was easy to distinguish the central layer of the cells that had darker elongated nuclei (the putative supporting cells) and the basal layer of the cells that had brighter oval nuclei (probably represent the precursors of the sensory cells). The nuclei of the supporting cells remained significantly darker up to the end of stage VI. It is worth mentioning that the cells that had elongated nuclei were never observed inside the forming columns. Thus, we do not assume that the precursors of bipolar neurons occur only within the columnar agglomerations during embryogenesis.

On the basis of our and other embryological data [23, 28] as well as studies of the adult histology of the VNO in Squamata [8, 19, 44, 45], we suggest that the most important step in the process of the columns formation is the undulation of the basal lamina. The invagination of this structure toward the VNO lumen probably creates the spaces for the intrusion of developing connective tissue and capillaries. In this way, compartments that separate the columns may arise. In fact, formation of columns begins in the basal part of the sensory epithelium. Alternatively, intrusion of the blood vessels into the sensory epithelium may cause an invagination of the basal lamina. There is a substantial difference in our interpretation and the one provided by Holtzman and Halpern [55, 56] in relation to the localization of the border between the supporting cells and the layer of the precursors of bipolar neurons. In our view, the adult-like condition of the sensory epithelium of the VNO is attained not only by simple change of the proportion of the supporting cells to the precursors of neurons, but also by gradual incorporation of the precursor of neuron located outside the column (more apically) into these structures. Nevertheless, decreasing number of supporting cells rows in relation to the undifferentiated and bipolar cell layer during embryogenesis of the vomeronasal sensory epithelium, leading to the adult-like condition of the single row of the supporting cells, seems to be common at least in snakes and mammals [8, 55, 56, 84, 85]. This may be due to decreasing number of the stem cells in the supporting cell layer ([56], see below) in parallel with the increasing size of the VNO and the pressure exerted on this layer by the developing columns. The latter could push the supporting cells and move them to the apical parts of the sensory epithelium.

The satellite cells (present under the basal lamina and surrounding the columns) may be important in establishing the final structure of the columns. In fact, these cells were visible at least from stage VIII in which the columns were well developed and extended almost across the entire thickness of the basal layer. However, the source of these cells is unknown.

Wang and Halpern [8] suggested that the sensory epithelium columns in snakes are not only a structural but also a dynamic unit for the postnatal neurogenesis. According to this view, changes in cell morphology at different levels of the epithelium are results of a continuous process of cell proliferation, differentiation, maturation and aging. During this process, cells from the basal part of the columns migrate toward the apical region. In other words, the undifferentiated cells represent the stem cells of the VNO neurons. Finally, degenerated neurons are extruded into the VNO lumen. We were able to find connections between the columns and the part of the basal layer that was undifferentiated into columns in our material. This could create a pathways through the connective tissue for the dendrites and extrusion of degenerated bipolar neuron. These “connection zones” were present between the columns and parts of the basal layer with were undifferentiated into the columns up to stage X. The connections probably pass through the apertures of the membranous platform that is created by the undulated basal lamina (see [8, 44]).

Wang and Halpern suggested that a population of undifferentiated cells for the replacement of the supporting cells may also be present in the embryonic vomeronasal epithelium in snakes [8]. They proposed that these cells could be located in the periphery of the columns or in the transitional region between the mushroom body and the sensory epithelium. However, there is some evidence that the precursors of the supporting cells are located within the layer of the supporting cells. Holtzman and Halpern [56] suggested this explanation on the basis of their autoradiography studies of the *Thamnophis* embryos. Buchtova et al. [29] found proliferating cells, using immunohistochemistry methods, in the apical part of the sensory epithelium at an early embryonic development stage of *Python*. The common mitotic figures in the peripheral laver, that consisted of few nuclei, were observed previously in snakes by Parsons [23]. The same was observed in our study and we noted this pattern in the early embryogenesis (developmental stages II-V).

We consider the condition of the sensory epithelium at stage X to be of an a adult-like morphology –although some changes, e.g. cell size and packing density in the sensory epithelium, may occur during the postnatal development [55]. In fact, it seems that the VNO is functional at birth in snakes and other squamate reptiles, and it was suggested that it may be functional even in their embryonic life [85]. On the basis of the light microscopic analysis, it was found that precursors of the supporting cells, neurons and undifferentiated cell layer are not clearly distinguishable in rat until its birth, and that some changes occur during the first 16 days after that. Thus, it was suggested that the VNO of rat is able to receive chemical stimuli at the end of the second week after birth [84]. Although the study of the metabolic activity of the accessory and main olfactory bulb may indicate that the VNO is functional in the embryos of rats and snakes, lack of the cytochrome oxidase staining in the main olfactory bulb of the snake embryos until they hatch may suggest at least differences in importance of the VNO in mammals and snakes during the neonatal period [85].

### Phylogeny vs. recent ecological interactions

When observing the embryonic development of the grass snake, we were able to note different levels of the anatomical or histological complexity of the VNO and associated structures (Fig. 10). The majority of the embryological –histological or anatomical data –for Squamata in respect to the analysed structures are available only for snakes. Our study indicates that the embryology of the analysed structures is very similar in the grass snake and other species of snakes that were studied, and the differences in descriptions seem to be results of different interpretations (e.g. concerning the border between the precursors of the supporting cells and bipolar neurons in the sensory epithelium of the VNO, or the connection of the early embryonic VNO with certain part of the nasal cavity). Similarities are not surprising due to the fact that the most of the study has been made for Caenophidia [23, 28, 55, 56]. Nevertheless, our 3D images allowed us to come up with detailed descriptions of the analysed structures, and thus, provide important data for future comparative embryology studies within Squamata. In fact, some of our findings on the grass snake embryo (e.g. the general morphology of the VNO including the ventral channel and the VNO duct, the morphology and origin of the anterior diverticula of the lacrimal duct, the shape of the cartilage of the mushroom body) were not comparable with other studies due to lack of similar reconstructions. Despite the fact that the conclusion about the embryonic development similarity in snakes may be affected by comparing mostly one clade, it appears that the early embryology of the squamate is truly evolutionarily conservative and that the direct connection between the VNO and the nasal cavity, as well the association of the anterior end of the lacrimal duct with the Choanengang, is a common state in the embryogenesis of lizards and snakes [22–24, 28, 29, 31]. This also resemble the condition present in adult tuatara (*Sphenodon punctatus*) [22, 23, 81], which is a member of Rhynchocephalia, commonly considered to be a sister taxon to Squamata [86–89]. In fact, embryonic development is, to some degree, evolutionarily conservative, and thus, provides a great source of information about evolutionary history of certain taxa (e.g. [90–92]). Our study provides data for comparative studies between the embryonic structures of the grass snake and the adult structures of other squamates or *Sphenodon*. Some similarities can be pointed out and certain assumptions about the homology can be made (see Fig. 10). However, few assumptions and comments should be noted in this comparison:

1. extant taxa can be considered only an approximation of ancestral conditions and embryonic development is not only under the phylogenetic but also under selective pressure (e.g. [93]); thus some modifications, in comparison to the true ancestor of the grass snake, are obvious,
2. similarities (presumptive homologues) can be distinguished only in some (not all) of the analysed structures, and, in some cases, at certain developmental stages homologues of different taxa can be observed simultaneously (e.g. the presence of the gekkotan, typical squamate and scolecophidian conditions at the developmental stage VI), which may be results of heterochrony,
3. in the description legend, we provide only a simplified phylogenetic tree, and thus only some groups of squamates are considered; additionally, it reflects the morphological view of phylogeny due to the basal position of Iguania (see below);
4. in our comparison, we excluded the ophidian-like forms of non-ophidian squamates due to the presumptive presence of their convergent evolution (see below),
5. the clades: Scolecophidia and Henophidia may be paraphyletic (e.g. [89, 94]),
6. due to lack of sufficient data in literature, some final conditions (observed in the last analysed stage) were not considered as a caenophidian-like or other squamate-like; for the same reason many levels of the anatomical or histological complexity are incomparable in the phylogenetic context.

Unfortunately, there seems to be lack of sufficient data for comparative studies within adult snakes and on comparisons of the sensory epithelium of the VNO within adult members of Squamata. As far as the first one is considered, it is difficult to compare the conditions of the VNO and associated structures among different ophidian families. There is, however, some exception from this and we can define the more plesiomorphic (the scolecophidian or henophidian) and apomorphic (the caenophidian) condition. The first is related to the character of the connection between the posterior end of the lacrimal duct and the Harderian gland [40, 57]; the second is related tothe morphology of the cupola Jacobsoni [32, 34, 42,43,]. Despite the fact that close connection of the posterior end of the lacrimal duct with the Harderian gland, and the direct connection of the anterior end of the lacrimal duct with the VNO duct as well the condition in which the vomeronasal nerve pierces the vomer (or the septomaxilla in some scolecophidians) seem to constitute synapomorphies of snakes, these are not unique for this clade. Some snake-like features (scolecophidians, henophidians or caenophidians) are present in other members of the non-ophidian squamates: dibamids, some amphisbaenids, pygopodids, monitor lizards and Xantusiidae. According to some molecular studies (e.g. [86, 87, 89]), all of them are rather distantly related taxa. Thus, presence of the snake-like conditions in these representatives seems to be considered as a result of their convergent evolution. Some of these may be an adaptation to the underground life (dibamids, amphisbaenids, pygopodids). Indeed, the view that snakes evolved probably from a burrowing or semi-burrowing ancestors is supported by morphological [95, 96] and molecular data [97]. According to the current knowledge, it seems that the main groups snakes are rather evolutionarily conservative in respect to the anatomy of the VNO and associated structures. However, the route of the lacrimal duct exhibits variation [27] and it may be associated with different ecological factors and the snout morphology. The latter as the whole head morphology may exhibit great variation even within one snake family as a result of an adaptation to prey type, habitat use, use of burrows or activity pattern [98].

According to the morphological view of phylogeny, Squamata is divided into two main groups – Iguania and Scleroglossa. The latter is subdivided into Gekkota and Autarchoglossa (e.g. [88, 99]). Autarchoglossans are considered to be a group in which a greater degree of chemical prey discrimination abilities evolved in the context of vomerolfaction (e.g. [71, 88, 99, 100]). In contrast to iguanian lizards, the tongue of autarchoglossans, especially such forms as in monitor lizards and snakes, appears to be specialised for vomerolfaction. The VNO in Iguania has been described as generally poorly developed [31, 71]. However, despite these facts, the vomeronasal chemoreception may still be important in some iguanians [101]. Indeed, there is no reliable measure of the vomeronasal chemoreception abilities of certain squamate taxa and, as we have mentioned before, there is still not much information about the histological diversity of the sensory epithelium of the VNO in non-ophidian squamates. In the grass snake embryos, we observed a gradual growth in the height of the sensory epithelium columns and widening of the spaces between them. It is possible that these different levels of the development of the sensory epithelium columns can be found in different lizard taxa. In fact, presence of these columns has been noted only in some adult lizards without any detailed descriptions [23, 45].

In contrast to morphological reconstruction of phylogeny analyses, molecular analyses revealed that Iguania is highly nested within Squamata, and thus, Scleroglossa is not a monophyletic group (e.g. [83, 86, 89]). In the light of this view on phylogeny, iguanian lizards exhibit great levels of homoplasy in different anatomical systems. Our study does not provide support for any of the phylogenetic topology. Although, we observed the iguanian-like condition with respect to the connection between the choanal groove and the VNO duct, the same condition is present in Gekkota, which are one of the most basal squamates according to molecular studies. This similarity between the geckoes and Iguania is not surprising, and historically both were combined into one group, Ascalabota [102]. However, it was a non-monophyletic group, formed on the basis of plesiomorphic traits (e.g. [71, 88]). We are not able to evaluate the homology between the direct connection of choanal grove and the VNO duct, observed in the grass snake embryo at the developmental stage IV, and the same condition present in Iguania and Gekkota. However, the character of the lacrimal duct and the choanal groove connection (developmental stages IV and VI) is rather gecko-like, and thus, the VNO duct which is confluent with the choanal groove seems to be represents also gecko-like condition. Nevertheless, studies on the other squamates embryology are necessary to understand the true evolutionary relationship between the main groups of Squamata.

## Conclusions

The main ophidian taxa (Scolecophidia, Henophidia and Caenophidia), just like other squamates clades, seem to be evolutionarily conservative at some levels with respect to the VNO and associated structures morphology. Because of that it was possible for us to homologize certain embryonic levels of the anatomical and histological complexity observed in the grass snake with the adult conditions of certain groups of Squamata. The main events of the embryonic development described here appear to reflect, at least to some degree, the evolutionary history of the gradual transition of squamates from basal taxa relying mainly on visual and olfactory cues to becoming the vomerolfaction specialists. The present paper shows the embryonic development of the VNO and associated structures in the grass snake in detail, utilizing both histology and 3D reconstructions. Our descriptions offer material useful for future comparative studies of Squamata, both at their anatomical and histological levels. The latter should include an ultrastructural and immunohistological study of the VNO sensory epithelium aiming at revealing the role and the process of the columns formation. Comprehensive comparative analyses of squamates are necessary to understand better the evolution of this one of the most diverse vertebrate group.

## Additional file

Additional file 1: The transverse sections through the choanal region of the grass snake embryos at different developmental stages Abbreviations: *AOR* Antorbitalraum, *CH* choana, *CHG* choanal groove, *DDNP* dorsal communication of the nasopharyngeal ducts, *DNP* nasopharyngeal duct, *FDNP* fusion of the nasopharyngeal ducts, *LD* lacrimal duct, *MPT* medial palatal trough, *OC* oral cavity, *T* tongue. Scale bars 100 μm. (TIF 13612 kb)

## Abbreviations

3D: Three-dimensional
VNO: Vomeronasal organ
VNS: Vomeronasal system
