# Supplementary figures and images for: Embryology of the VNO and associated structures in the grass snake Natrix natrix (Squamata: Naticinae): a 3D perspective
Source: Front Zool. 2017 Jan 13;14:1. doi: 10.1186/s12983-017-0188-y (PMC5237294; doi:10.1186/s12983-017-0188-y)

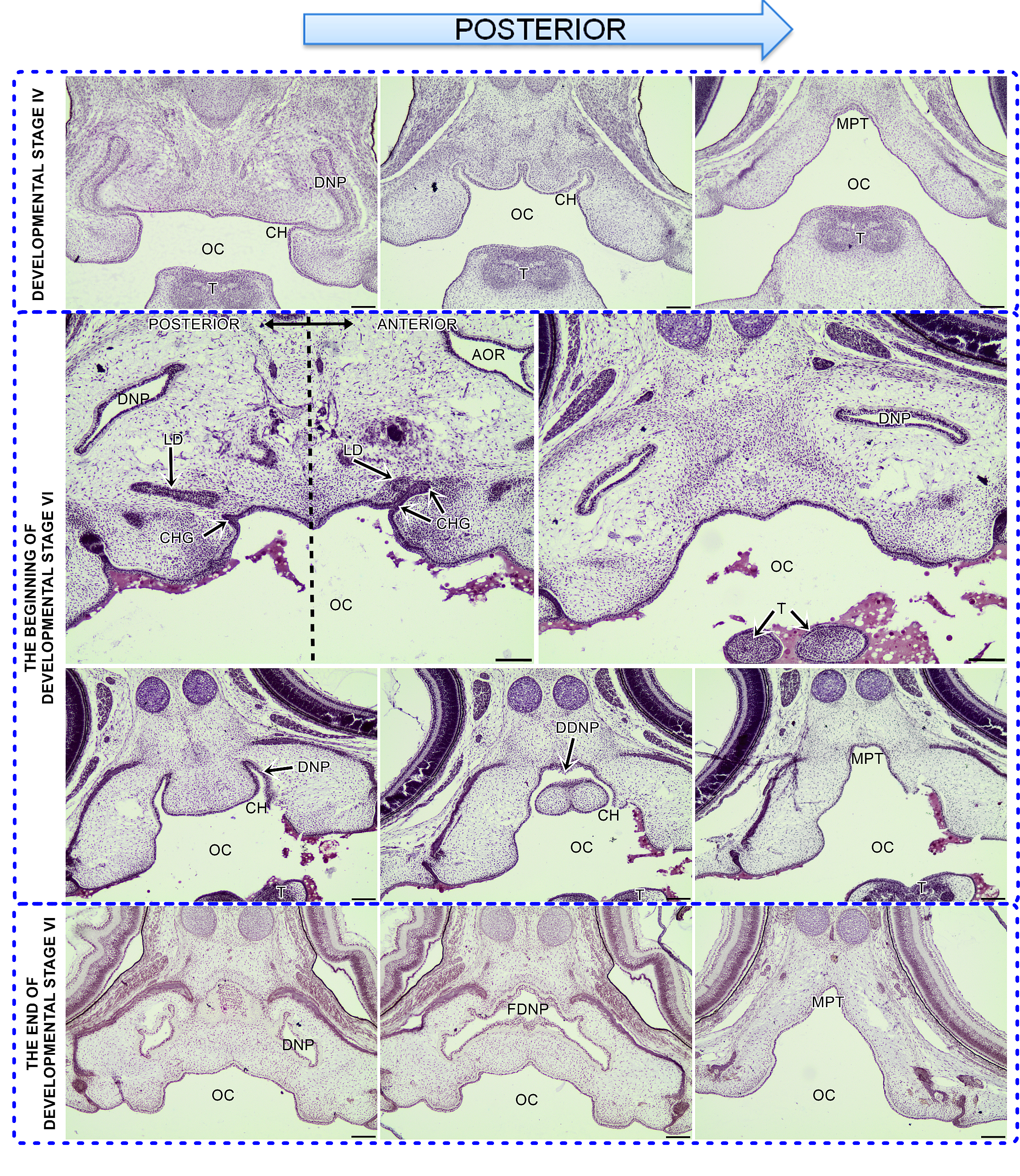

Supplement: Additional file 1: — The transverse sections through the choanal region of the grass snake embryos at different developmental stages Abbreviations: AOR Antorbitalraum, CH choana, CHG choanal groove, DDNP dorsal communication of the nasopharyngeal ducts, DNP nasopharyngeal duct, FDNP fusion of the nasopharyngeal ducts, LD lacrimal duct, MPT medial palatal trough, OC oral cavity, T tongue. Scale bars 100 μm. (TIF 13612 kb) [file 12983_2017_188_MOESM1_ESM.tif]
